# Supplementary material for: Diverse regulatory pathways modulate bet hedging of competence induction in epigenetically-differentiated phase variants of Streptococcus pneumoniae
Source: Nucleic Acids Res. 2023 Sep 27;51(19):10375–94. doi: 10.1093/nar/gkad760 (PMC10602874; doi:10.1093/nar/gkad760)
Supplement: gkad760_Supplemental_Files [file gkad760_supplemental_files.zip › RMV7_SOM_NAR_revision2_clean.docx]

**Diverse regulatory pathways modulate bet hedging of competence induction in epigenetically-differentiated phase variants of *Streptococcus pneumoniae***

Min Jung Kwun, Alexandru V. Ion, Marco R. Oggioni, Stephen D. Bentley, Nicholas J. Croucher

**Supplementary materials**

Text S1-S5

Supplementary References

Figures S1–S49

Tables S1-S7 (legend only for Table S5)

**Supplementary Text**

**Text S1: Quantification of 3’,5’-cAMP production**

Quantification of 3’,5’-cAMP used the Cyclic AMP XP^®^ assay kit (Cell Signalling Technology). Samples were harvested in the exponential (OD_600_ of 0.2) and stationary (OD_600nm_ of 0.5) phases of statically-grown *S. pneumoniae* cultures. *E. coli* DH5⍺ was grown in 25 mL Luria-Bertani media (Sigma-Aldrich) at 37 °C, shaken at 250 revolutions per minute, and samples were harvested in the exponential (OD_600_ of 0.3) and stationary (OD_600nm_ of 1.0) phases. Cells were pelleted through centrifugation at 3,220 *g* for 10 min. The cell pellets were re-suspended in 850 μL of the kit’s lysis buffer and 150 μl of lysozyme (10 mg mL^-1^), followed by incubation at 35 °C for 20 minutes. Cells were pelleted by centrifugation at 9,500 *g* for 5 minutes. The supernatants were collected, and the cell pellets were resuspended in 400 μl phosphate-buffered saline. The protein concentrations of all the collected samples were adjusted to 400 μg mL^-1^ using the Qubit protein broad range assay kits (Qiagen). The Cyclic AMP XP^®^ kit ELISA plates were prepared according to the manufacturer’s protocols, and a 50 μl sample of supernatant from each tested culture was loaded in each well. The OD_450_ was measured using the FLUOstar Omega plate reader. Concentrations of 3’,5’-cAMP were calculated using a standard curve, according to the manufacturer’s instructions.

**Text S2: Statistical analyses of transformation efficiency assays**

**Analysing the effect of Ca^2+^ ion supplementation**

A logistic curve was used to analyse the relationship between transformation efficiency (in transformants per 10^4^ colony forming units), *t*, and the concentration of the CaCl_2_, *x*. The fitted function was:

$$t=\frac{a}{1+e^{-b(x-c)}}$$

The values of the variables *a*, *b* and *c* were estimated using the Levenberg-Marquardt nonlinear least-squares algorithm in the minpack R package (1), using the starting values of 10 (increased to 500 for *S. pneumoniae* R6), 1 and 0.5, respectively. The confidence intervals were calculated through refitting the function to 999 bootstrapped samples using the car R package (2).

**Estimation of the frequency of competence induction**

The calculation of the proportion of cells in which competence was induced required the transformation of cultures with two independent selectable markers. The frequency of transformation with each single marker, *f*, can be expressed as the product of the proportion of pneumococci that are competent for transformation, τ, and the probability of acquisition of each marker: *p*_rif_ for rifampicin, and *p*_kan_ for kanamycin. Hence the product of these two transformation efficiencies, *P*_double_, is:

$$P_{double}=\left( \tau p_{rif} \right)\left( \tau p_{kan} \right)=\tau^{2}p_{rif}p_{kan}$$

The observed frequency of double mutants, *O*_double_, can be expressed as the product of the probability a cell is competent for transformation, *p*_rif_ and *p*_kan_:

$$O_{double}={\tau p}_{rif}p_{kan}$$

Therefore the proportion of bacteria that are competent for transformation within a culture can be calculated as:

$$\frac{P_{double}}{O_{double}}=\frac{\tau^{2}p_{rif}p_{kan}}{{\tau p}_{rif}p_{kan}}=\tau$$

Note that if all cells in the culture are transformable, then *P*_double_ = *O*_double_, whereas an excess of double mutants over the frequency expected from the single mutant frequencies is evidence that only a subset of the population is competent for transformation.

**Text S3: Details of mutant construction**

Disruption of genes for directed mutagenesis required the PCR amplification of 0.8-1 kb regions flanking the gene of interest, using the oligonucleotide sequences listed in Table S2. For each gene, the upstream region was amplified with the oligonucleotides labelled with the gene name and the suffixes “Up_For” and “Up_Rev_ApaI”, with the latter adding an *Apa*I site to the 3’ of the amplicon. The downstream region was amplified with the oligonucleotides labelled with the gene name and the suffixes “Down_For_BamHI” and “Down_Rev”, with the former adding an *Bam*HI site to the 5’ of the amplicon. The corresponding antibiotic resistance markers were amplified with oligonucleotides that added flanking *Bam*HI and *Apa*I restriction enzyme sites: Janus_For_ApaI and Janus_Rev_BamHI for the Janus cassette, or Cat_For_ApaI and Cat_Rev_ApaI for the *cat* chloramphenicol resistance marker (3). PCR products were digested with the appropriate restriction enzymes (Promega) at 35 °C for 2-4 hours, and then ligated to the appropriate antibiotic marker using T4 DNA ligase (Invitrogen). Ligation mixtures were used as templates to amplify correctly-ligated constructs through PCR using the “Up_For” and “Down_Rev” oligonucleotides, which generated the amplicons used for mutagenesis through transformation of competent pneumococcal cells.

Restoration of genes disrupted using the Janus cassette depended on all genotypes in which mutations were generated originally being resistant to streptomycin (Table S1), owing to a mutation in *rpsL* (4). Mutants were isolated in which the Janus cassette restored susceptibility to streptomycin. This enabled the Janus cassette to be removed through transformation with PCR amplicons, encoding the intact gene, generated from the original genotypes with the “Up_For” and “Down_Rev” oligonucleotides, using selection for streptomycin-resistant cells. As intragenomic recombination generates false positive streptomycin-resistant genotypes independently of transformation, identifying true positive recombinants in which genes were restored through eliminating the Janus cassette was only feasible in the more transformable RMV7_rare_ variant. PCR amplification was used to ensure the complete removal of the cassette.

The exception to this approach was the construction of RMV7 *tvr*_rare_::Janus and RMV7 *tvr*_domi_::Janus. Both genotypes derived from the RMV7_wt_ *tvr*::*cat* mutant, which was transformed with DNA from RMV7_domi_ or RMV7 *tvr*_rare_ *tvrR*::Janus, followed by selection on plates supplemented with kanamycin. The RMV7 *tvr*_rare_ *tvrR*::Janus genotype was the original locked phase variant from which RMV7_rare_ was derived (3), through removal of the Janus cassette. Using RMV7 *tvr*_rare_::Janus as the donor in this transformation allowed kanamycin selection to be used to isolate transformed genotypes that had acquired the modified *tvr* locus. Notably, the original isolation of different phase variants through disruption of *tvrR* used different constructs, and therefore RMV7_domi_ was locked through the replacement of 681 bp from the 3’ end of *tvrR*, whereas RMV7 *tvr*_rare_ *tvrR*::Janus was locked through the replacement of 267 bp from the 5’ end of the gene (Fig. 1). Hence the comparison of RNA-seq data across the two variants identified differential levels of sequence read mapping to *tvrR* (Fig. 2).

**Text S4: Details of quantitative PCR analyses**

All RNA samples were extracted using the SV Total Isolation System (Promega) according to the manufacturer’s instructions, with the exception that the initial extraction of RNA required treating cell cultures with 480 μL 30 mg mL^−1^ lysozyme (Promega) for 30 min at 35 °C. This method includes a DNase I treatment step.

The quality of the extracted RNA was assessed visually using agarose gel electrophoresis, to identify any degraded samples. The A_260_/A_280_ ratio was measured with a Nanodrop ND-1000 spectrophotometer (Thermo Fisher Scientific), to assess levels of protein contamination. This device was also used to quantify the concentration of RNA in the sample.

This enabled 0.2 μg samples of pure, undegraded RNA to be individually treated with amplification-grade DNase I (Invitrogen) according to the manufacturer’s instructions. This 0.2 μg of DNase-treated RNA was then used in reverse transcription reactions using the First-Strand III cDNA synthesis kit (Invitrogen). Each reaction used 100 units of the SuperScript III reverse transcriptase, 1 μL of 100 μM random hexamer primers (Thermo Fisher Scientific) and 1 μL of 10 mM dNTP mix (Bioline). The final concentration of Mg^2+^ ions in the buffer was 1.2 mM. Annealing of oligonucleotides occurred during a 5 min incubation at 25 °C. The reaction was then initiated through incubation at 50 °C for 30 min, followed by a further 30 min incubation at 55°C. Finally, the reverse transcriptase was inactivated by heating at 70 °C for 15 min. These concentrated cDNA samples were stored at -80 °C.

Genomic DNA of RMV7_wt_ was diluted to copy numbers of 3x10^3^, 3x10^4^, 3x10^5^ and 3x10^6^, using concentration measurements from the NanoDrop spectrophotometer. These samples were used for generating standard curves for each tested gene, and the *rpoA* reference gene.

Primers for qPCR were designed to generate amplicons that were 150-200 bp in length using Primer3 (<https://primer3.ut.ee/>). Their sequences are provided in Table S2. cDNA was diluted in a 1:25 ratio with DNase-free and RNase-free water (Qiagen). Each reaction used 3.75 μL of DNA template (either genomic DNA or cDNA), 0.75 μL of 10mM forward and reverse primer solutions (Invitrogen),7.5 μL of PowerUp™ SYBR™ Green Master Mix (Thermo Fisher Scientific) and 2.25 μL of DNase-free and RNase-free water. Hence the total reaction volume was 15 μL.

Reactions were run using MicroAmp^TM^ Optical 96-well reaction plates (Thermo Fisher Scientific) and the QuantStudio™ 7 Flex Real-Time PCR System (Applied Biosystems). The mixtures were initially heated to 50 °C for 2 min. Subsequently, 40 amplification cycles were run, each consisting of denaturation at 95 °C for 15 s, followed by annealing and elongation at 60 °C for 1 min. The purity of the amplicon at the end of the reaction was assessed through a melt curve analysis.

All quantifications were measured relative to the abundance of *rpoA*, which has been validated to be an appropriate reference gene in multiple bacterial species (5–7). Standard curves for each tested gene, and the reference *rpoA* gene, were generated independently on each plate. The success of the reaction was confirmed by a strong correlation between Ct values and DNA concentrations in the standard curve reactions (*R*^2^ being close to 1).

Each technical replicate was conducted in a separate well, but used the same cDNA template. Analyses that were validating inferences from RNA-seq or previously-established regulatory pathways used two biological replicates, each of which was analysed with three technical replicates. Other experiments used three biological replicates, each of which was again analysed with three technical replicates.

To estimate the difference in gene expression following the disruption of PRCI*_dnaN_*_+_*_att_*, a linear mixed effects model was fitted jointly to the technical and biological replicate measurements of the levels of chaperone gene expression shown in Fig. 4C using the R package lme4 (8). The model had the form:

$$Expression\sim Gene*PRCI+\left( 1 | Gene:{Replicate}_{Biological} \right)+1$$

The term “$PRCI$” is a binary factor defining whether PRCI*_dnaN_*_+_*_att_* was present in the genotype being assayed. Therefore the fixed effect $Gene*PRCI$ estimates the effect of removing the PRCI on each gene. The random effect $\left( 1 | Gene:{Replicate}_{Biological} \right)$ corrects for the structure in the data resulting from the three technical replicate measurements of each of three biological replicate samples. Calculation of the associated confidence intervals used the R package multcomp (9).

To quantify the dynamics of *tvr* loci during culture, three independent passages of RMV7_wt_ in liquid media were each initiated through inoculation with a single colony. Each subsequent 24 h passage was inoculated with 10 μL of the previous culture. Genomic DNA was extracted from the initial culture, and after four and eight days of passage, using the Wizard Genomic DNA purification kit (Promega). Quantitative PCR was used to measure the frequency of each distinguishable allele of the *tvr* locus, using triplicate technical replicates on each sample, as described previously (10). The absolute copy numbers of each allele were then estimated using standard curves, thereby enabling the *tvr*_domi_:*tvr*_rare_ ratio to be calculated.

**Text S5: Bioinformatic and genomic analyses**

To identify distinguishing polymorphisms, the RMV7_domi_ and RMV7_rare_ sequences were aligned with nucmer (11). The overall distribution of methylation motifs was calculated using DistAMo (12). The calculation of distances between coding sequences and motifs used biopython (13).

The *S. pneumoniae* RMV7 TfoX and YjbK proteins were aligned to orthologues from *H. influenzae* Rd and *V. cholerae* ATCC 39315 using Muscle (14). The predicted structures were retrieved from the AlphaFoldDB (15).

Proteins containing the TfoX N-terminal domain were identified using EMBL SMART (16). These amino acid sequences were aligned with MAFFT (17), and a phylogeny generated with Fasttree2 (18) using default settings. Proteins were assigned to bacterial Families using the NCBI Taxonomy (19).

**Supplementary References**

1. Elzhov,T. V, Mullen,K.M., Spiess,A.-N. and Bolker,B. (2016) minpack.lm: R Interface to the Levenberg-Marquardt nonlinear least-squares algorithm found in MINPACK, plus support for bounds.

2. Fox,J. and Weisberg,S. (2019) An {R} Companion to Applied Regression Third. Sage, Thousand Oaks {CA}.

3. Kwun,M.J., Oggioni,M.R., De Ste Croix,M., Bentley,S.D. and Croucher,N.J. (2018) Excision-reintegration at a pneumococcal phase-variable restriction-modification locus drives within- and between-strain epigenetic differentiation and inhibits gene acquisition. *Nucleic Acids Res.*, **46**, 11438–11453.

4. Apagyi,K.J., Fraser,C. and Croucher,N.J. (2018) Transformation asymmetry and the evolution of the bacterial accessory genome. *Mol. Biol. Evol.*, **35**, 575–581.

5. Ritz,M., Garenaux,A., Berge,M. and Federighi,M. (2009) Determination of *rpoA* as the most suitable internal control to study stress response in *C. jejuni* by RT-qPCR and application to oxidative stress. *J. Microbiol. Methods*, **76**, 196–200.

6. Williams,M.L. and Ghanem,M. (2022) Evaluation of candidate reference genes stability for gene expression analysis by reverse transcription qPCR in *Clostridium perfringens*. *Sci. Rep.*, **12**, 19434.

7. Rocha,D.J.P., Santos,C.S. and Pacheco,L.G.C. (2015) Bacterial reference genes for gene expression studies by RT-qPCR: survey and analysis. *Antonie Van Leeuwenhoek*, **108**, 685–693.

8. Bates,D.M., Maechler,M., Bolker,B. and Walker,S. (2015) lme4: linear mixed-effects models using S4 classes. *J. Stat. Softw.*, 10.1088/1742-6596/43/1/292.

9. Bretz,F., Hothorn,T. and Westfall,P. (2010) Multiple Comparisons Using R Chapman and Hall/CRC.

10. Kwun,M.J., Oggioni,M.R., Bentley,S.D., Fraser,C. and Croucher,N.J. (2019) Synergistic Activity of Mobile Genetic Element Defences in *Streptococcus pneumoniae*. *Genes (Basel).*, **10**, 707.

11. Delcher,A.L., Phillippy,A., Carlton,J. and Salzberg,S.L. (2002) Fast algorithms for large-scale genome alignment and comparison. *Nucleic Acids Res.*, **30**, 2478–2483.

12. Sobetzko,P., Jelonek,L., Strickert,M., Han,W., Goesmann,A. and Waldminghaus,T. (2016) DistAMo: A Web-Based Tool to Characterize DNA-Motif Distribution on Bacterial Chromosomes. *Front. Microbiol.*, **7**, 283.

13. Cock,P.J.A., Antao,T., Chang,J.T., Chapman,B.A., Cox,C.J., Dalke,A., Friedberg,I., Hamelryck,T., Kauff,F., Wilczynski,B., *et al.* (2009) Biopython: freely available Python tools for computational molecular biology and bioinformatics. *Bioinformatics*, **25**, 1422–1423.

14. Edgar,R.C. (2004) MUSCLE: multiple sequence alignment with high accuracy and high throughput. *Nucleic Acids Res.*, **32**, 1792–1797.

15. Varadi,M., Anyango,S., Deshpande,M., Nair,S., Natassia,C., Yordanova,G., Yuan,D., Stroe,O., Wood,G., Laydon,A., *et al.* (2022) AlphaFold Protein Structure Database: massively expanding the structural coverage of protein-sequence space with high-accuracy models. *Nucleic Acids Res.*, **50**, D439–D444.

16. Letunic,I., Khedkar,S. and Bork,P. (2021) SMART: recent updates, new developments and status in 2020. *Nucleic Acids Res.*, **49**, D458–D460.

17. Katoh,K. and Standley,D.M. (2013) MAFFT multiple sequence alignment software version 7: Improvements in performance and usability. *Mol. Biol. Evol.*, **30**, 772–780.

18. Price,M.N., Dehal,P.S. and Arkin,A.P. (2010) FastTree 2–approximately maximum-likelihood trees for large alignments. *PLoS One*, **5**, e9490.

19. Schoch,C.L., Ciufo,S., Domrachev,M., Hotton,C.L., Kannan,S., Khovanskaya,R., Leipe,D., Mcveigh,R., O’Neill,K., Robbertse,B., *et al.* (2020) NCBI Taxonomy: a comprehensive update on curation, resources and tools. *Database (Oxford).*, **2020**.

**Supplementary Figures**

**B**

**A**

**Figure S1** Establishing the differing arrangements of the *tvr* loci in the RMV7 variants. (A) Schematic showing the binding site of oligonucleotides used for PCR amplification experiments. The forward primer binds an invariant site within *hsdM*. The four different reverse primers each bind a site specific to an individual TRD-encoding sequence, which are rearranged through the integration-excision activity of TvrR. (B) Separate PCR amplification experiments were conducted with the same conserved forward primer, and the four reverse primers, on RMV7_wt_, RMV7_domi_ and RMV7_rare_. In RMV7_domi_, the sequence encoding TRDiii forms part of the active *hsdS* gene, adjacent to *hsdM*, whereas the sequences encoding TRDII and TRDi are in the inactive, distal position. In RMV7_rare_, the sequence encoding TRDi forms part of the active *hsdS* gene, adjacent to *hsdM*, whereas the sequences encoding TRDII and TRDiii are in the inactive, distal position. In RMV7_wt_, which has an active TvrR, both arrangements are evident, as are alternative alleles in which the sequence encoding TRDII is proximal to *hsdM*. The size of products is shown relative to DNA Hyperladder 1kb (Bioline).

**Figure S2** Validation of differences in transformation between RMV7_wt_ and RMV7_rare_. (A) Frequency of spontaneous rifampicin resistant mutants in RMV7_wt_ observed after performing the transformation protocol, including the addition of CSP, in the presence and absence of exogenous DNA. The frequency of spontaneous rifampicin resistant RMV7_wt_ bacteria was very low in these experiments. This confirmed the majority of resistant bacteria observed in transformation experiments resulted from the uptake of exogenous DNA. (B) Both RMV7_wt_ and RMV7_rare_ were transformed, and the cells were plated on selective mediate after 20 h, rather than 2 h. This tested whether the induction of competence was slower, rather than lower, in RMV7_wt_. A significant difference between RMV7_wt_ and RMV7_rare_ was still observed, confirming the variation recorded in other experiments was not the consequence of delayed competence induction in RMV7_wt_. Significance between results is coded as: *p* < 0.05, *; *p* < 0.01, **; *p* < 10^-3^, ***; *p* < 10^-4^, ****.

RMV7_rare_

RMV7_domi_

RMV7_wt_

**Figure S3** Morphology of RMV7 variant colonies. Phase contrast microscopy was used to visualize colonies of RMV7_wt_, RMV7_domi_ and RMV7_rare_. All three had a consistent size: the three scale bars spanning the colonies shown at 10x magnification have lengths of 545, 502 and 589 μm, from top to bottom. This is consistent with all genotypes expressing a similar thickness of capsule. This distinguishes them from transparent and opaque phase variants, which exhibit distinctive colony morphologies.

**Figure S4** Testing the effect of mutations outside the *tvr* locus on variant transformation efficiency. (A) Violin plot showing the transformation efficiency of *phoB* knock out and knock in mutants of RMV7_wt_ and RMV7_rare_. Each point represents an independent transformation experiment. The violin plots summarise the transformation efficiency of each genotype, with a horizontal line representing the median transformation efficiency. The maintenance of a ~100-fold difference in transformation efficiency between RMV7_wt_ and RMV7_rare_ in the absence of *phoB* demonstrates the polymorphisms detected within this regulatory gene are unlikely to cause the phenotypic differences between RMV7_wt_ and RMV7_rare_. This is confirmed by the similarity of the transformation efficiency of RMV7_rare_ carrying the knocked in *phoB*_wt_ and *phoB*_rare_ alleles. (B) Violin plot showing the transformation efficiency of *pstS* knock out mutants of RMV7_wt_ and RMV7_rare_. The loss of the *pstS* gene, which contains a premature stop codon in RMV7_rare_, did not affect the difference in transformation efficiency between the variants.

3 kb

1 kb

0.4 kb

**B**

**A**

**
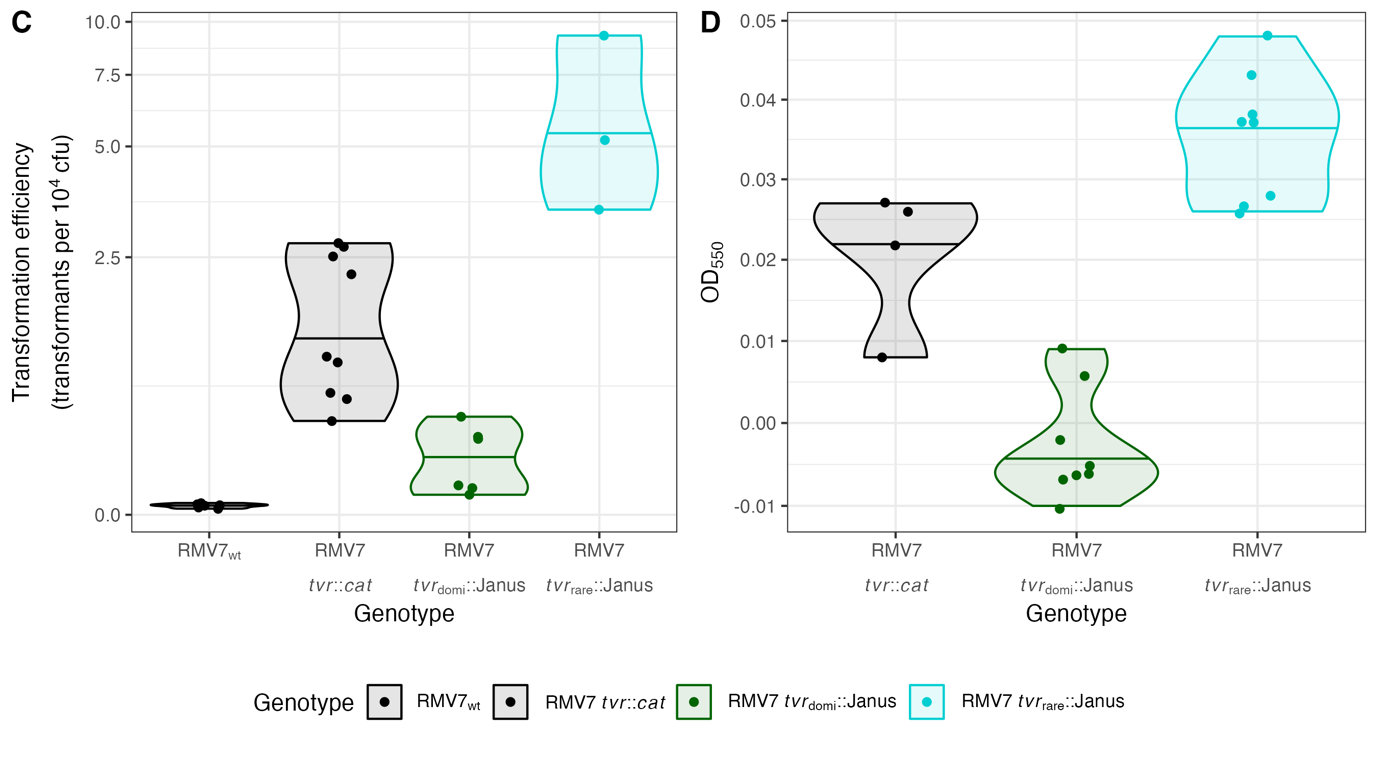
**

**Figure S5** Design of the RNA-seq and qRT-PCR experiments. (A) The *tvr* loci of RMV7_domi_ and RMV7 *tvr*_rare_::Janus, a progenitor of RMV7_rare_ that retained a Janus cassette inserted into *tvrR*, were introduced into a common RMV7_wt_ *tvr*::*cat* recipient. (B) The agarose gel shows the PCR amplicons generated with the primers described in Fig. S1, which demonstrates the intact *tvr* loci have been integrated into the recipients to generate RMV7 *tvr*_domi_::Janus and RMV7 *tvr*_rare_::Janus. The amplicon sizes are measured relative to DNA Hyperladder 1kb. (C) Violin plot showing the transformation efficiencies of genotypes from which RNA was extracted for RNA-seq analysis. These results demonstrated the insertion of the *tvr*_domi_::Janus and *tvr*_rare_::Janus loci replicated the divergence in transformation between RMV7_domi_ and RMV7_rare_. (D) Violin plot showing the thicknesses of biofilms formed by the genotypes from which RNA was extracted for RNA-seq analysis. These results demonstrated the insertion of the *tvr*_domi_::Janus and *tvr*_rare_::Janus loci replicated the divergence in biofilm thicknesses between RMV7_domi_ and RMV7_rare_.

**Figure S6** Density plots showing the inferred distribution of fragment sizes from mapping by Kallisto. The consistency of these distributions between samples, each labelled with its accession code (Table S4), suggests there should not be any bias introduced by differences in sequencing library preparation.

**Figure S7** Density plots showing the distribution of mean transcripts per million (tpm) values across genes for samples in different groups. (A) This density plot shows the mean tpm distribution is highly similar between samples originating between the two genotypes. (B) This density plot shows the mean tpm distribution changes slightly following the addition of CSP, which may represent the altered transcriptional patterns within the cell. However, the general shape of the distributions is similar, suggesting technical biases are unlikely to cause any observed differences between samples.

**Figure S8** Q-Q plot comparing the theoretical and observed distributions of the Wald test statistic across genes for the contrast of transcriptional patterns between RMV7 *tvr*_domi_::Janus and RMV7 *tvr*_rare_::Janus prior to the addition of CSP. The blue line shows the relationship expected under the null hypothesis of no difference in expression patterns. Each point represents a coding sequence. Points are coloured red if the null hypothesis can be rejected at a false discovery rate of 10^-3^, following a Benjamini-Hochberg correction for multiple testing. The Q-Q plot shows this threshold captures the major differences between the two genotypes.

**Figure S9** Q-Q plots comparing the theoretical and observed distributions of the Wald test statistic across genes for the comparison of transcriptional patterns following the addition of CSP. The plots are displayed as in Fig. S8. The panels show contrasts of (A) the 0 minute and 10 minute timepoints for RMV7 *tvr*_domi_::Janus, and (B) the 0 minute and 20 minute timepoints for RMV7 *tvr*_domi_::Janus; and (C) the 0 minute and 10 minute timepoints for RMV7 *tvr*_rare_::Janus, and (D) the 0 minute and 20 minute timepoints for RMV7 *tvr*_rare_::Janus.

**Figure S10** Heatmap illustrating the differences in transcriptional patterns across RNA-seq datasets. Each column or row of the heatmap corresponds to an RNA-seq dataset (Table S4), ordered by their similarity to one another, as illustrated by the dendrogram at the top of the figure. The rows beneath the dendrogram annotate the characteristics of each sample. The fill in each cell shows the pairwise Jensen-Shannon divergence. The matrix is symmetrical, with samples ordered identically across rows and columns, and dissimilarities of zero along the diagonal. These show the samples diverging most strongly from the rest are the post-CSP samples from the RMV7 *tvr*_rare_::Janus genotype.


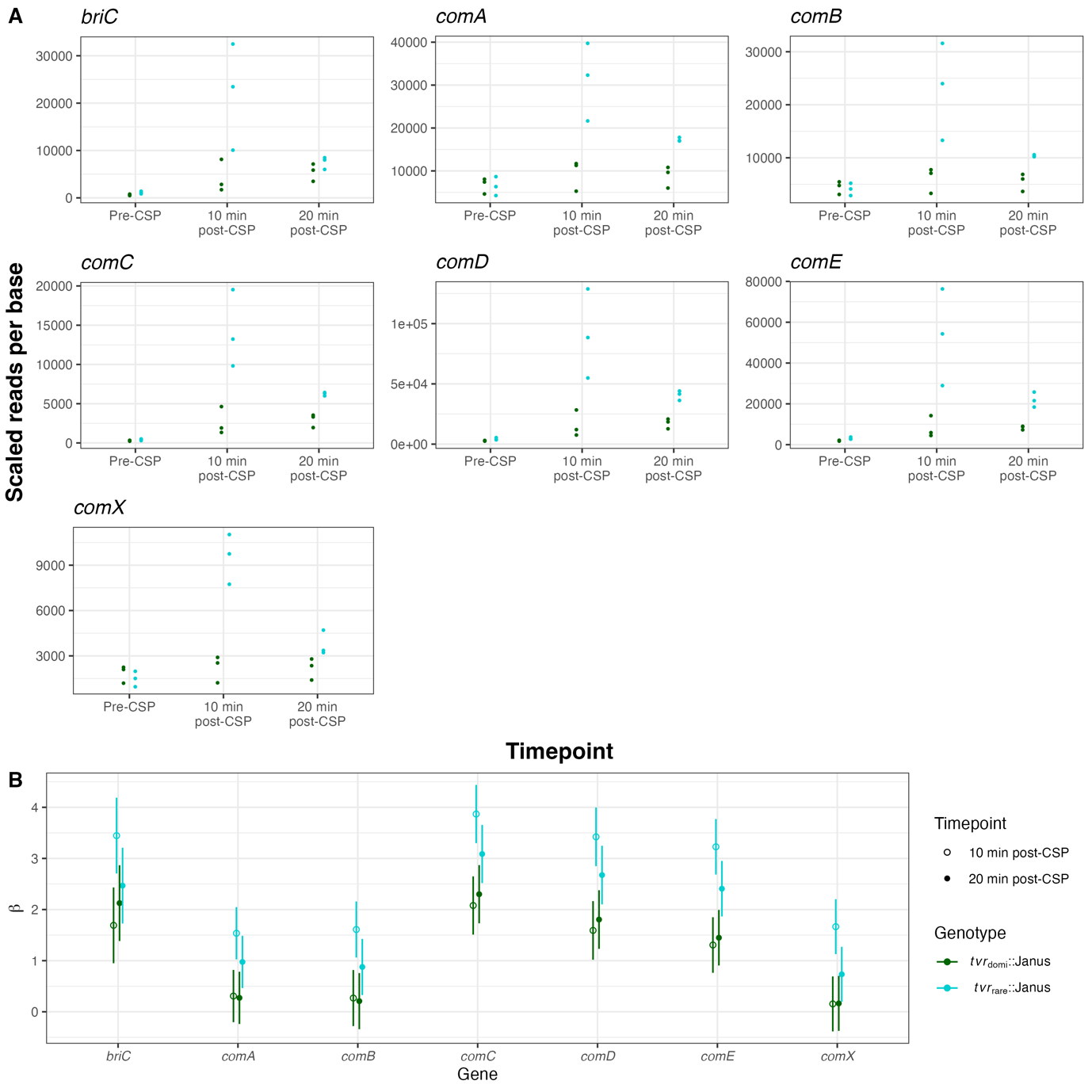


**Figure S11** Quantification of the expression of early competence genes using RNA-seq data. (A) Each plot shows the transcription of a different gene, in scaled reads per base, across the three assayed timepoints in the two variants. The colours indicate the variant in which expression was estimated. There are three biological replicates for each measurement. All these genes show a strong post-CSP upregulation in RMV7 *tvr*_rare_::Janus, with a weaker response in RMV7 *tvr*_domi_::Janus. (B) Estimates of β, the natural logarithm of the fold change, following the administration of CSP. The colours indicate the variant in which expression was estimated, and the fill of the point indicates the corresponding timepoint. The error bars show the 95% confidence intervals, estimated from the standard error of the β estimate.


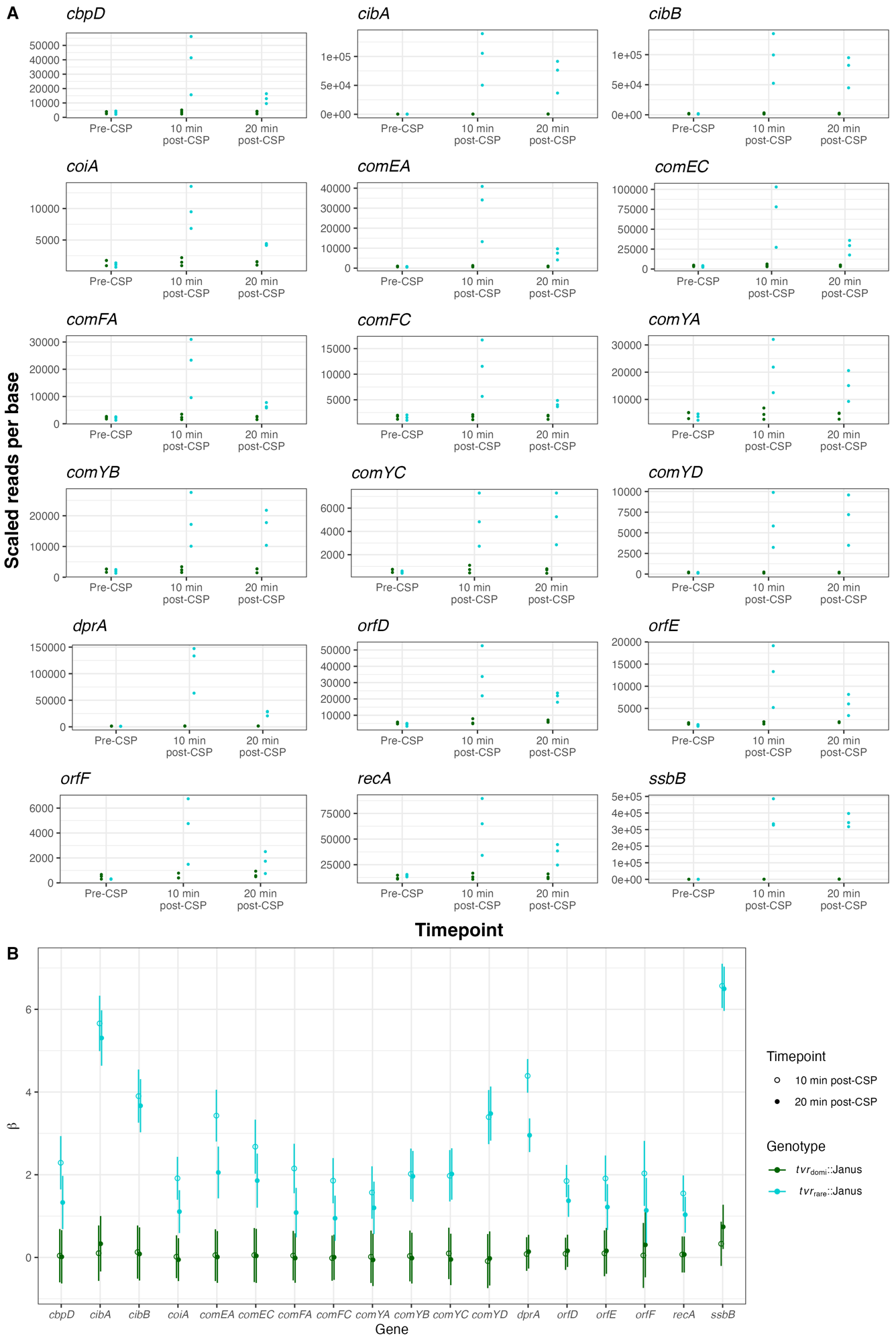


**Figure S12** Quantification of the expression of late competence genes using RNA-seq data. Data are displayed as in Fig. S11. A much stronger induction by CSP is observed in RMV7 *tvr*_rare_::Janus than RMV7 *tvr*_domi_::Janus.


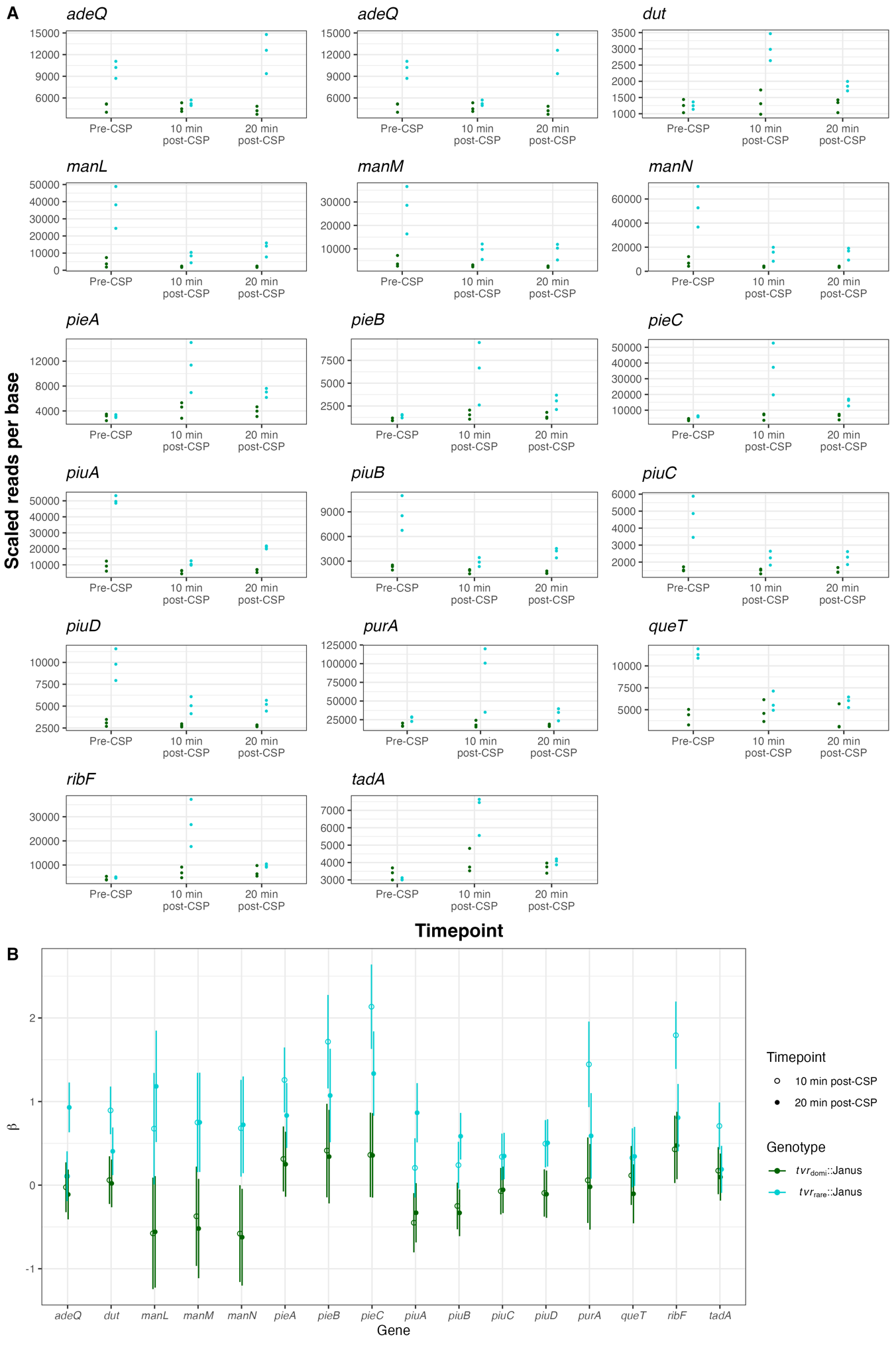


**Figure S13** Quantification of the expression of transporter and nucleotide metabolism genes using RNA-seq data. Data are displayed as in Fig. S11.

**Figure S14** Quantification of gene expression using qRT-PCR. Samples were collected from RMV7_wt_, RMV7_wt_ *tvr*::*cat*, RMV7_domi_ and RMV7_rare_ prior to the addition of CSP, and both 10 and 20 minutes after the addition of CSP, to mirror the RNA-seq experiment. Each plot corresponds to a specific gene. For each gene, datapoints were combined across three technical replicate measurements of each biological replicate. Data are positioned according to the timepoint at which they were sampled and coloured by the genotype of the cells from which they were collected. Expression was quantified as the abundance of transcripts relative to *rpoA* RNA. For each gene at each timepoint, two-tailed Wilcoxon rank sum tests were used to compare each genotype to RMV7_wt_. A Holm-Bonferroni correction for multiple testing was applied within each panel. Significance between results is coded as: *p* < 0.05, *; *p* < 0.01, **; *p* < 10^-3^, ***; *p* < 10^-4^, ****.


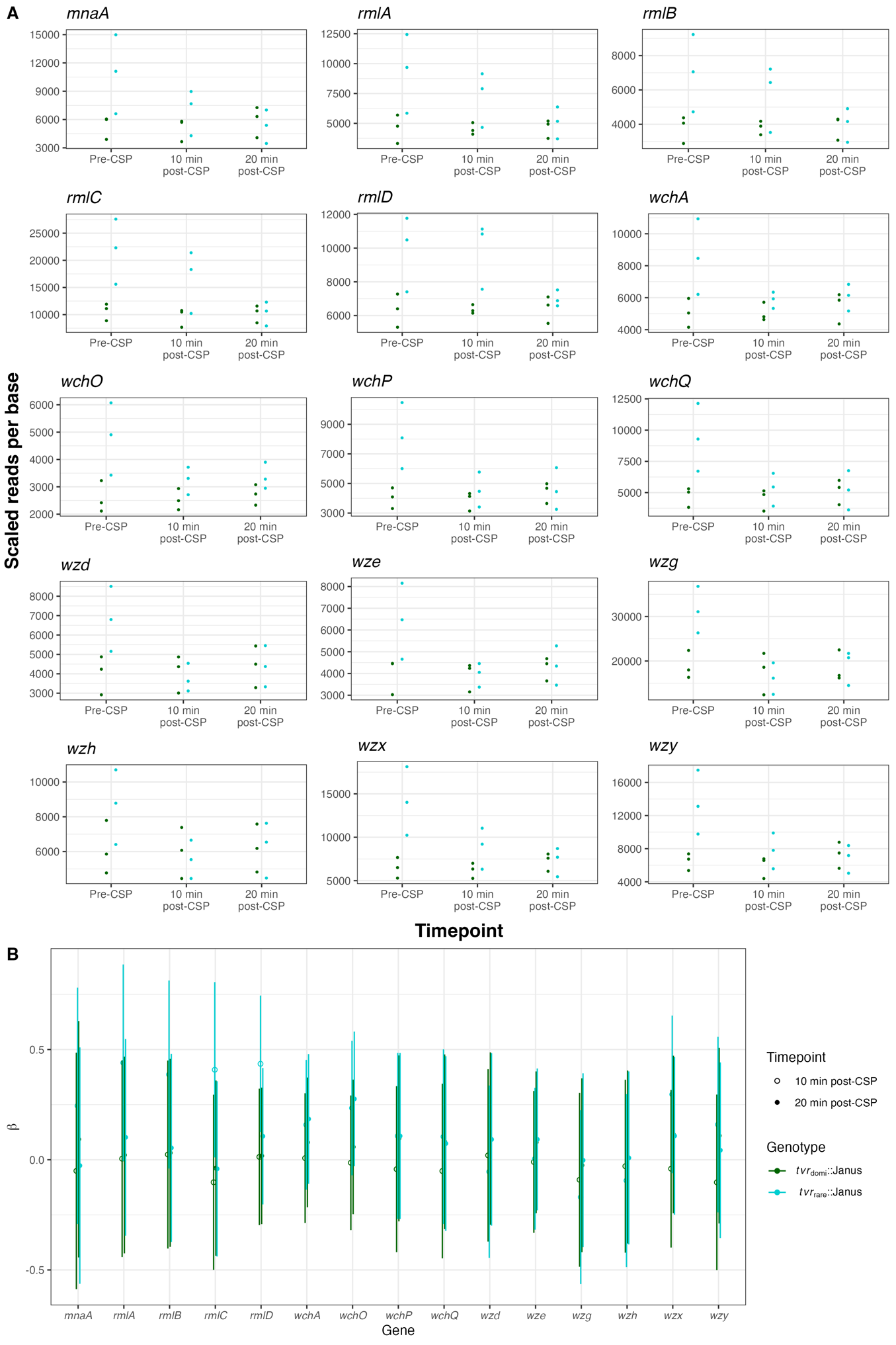


**Figure S15** Quantification of the expression of *cps* locus genes using RNA-seq data. Data are displayed as in Fig. S11.


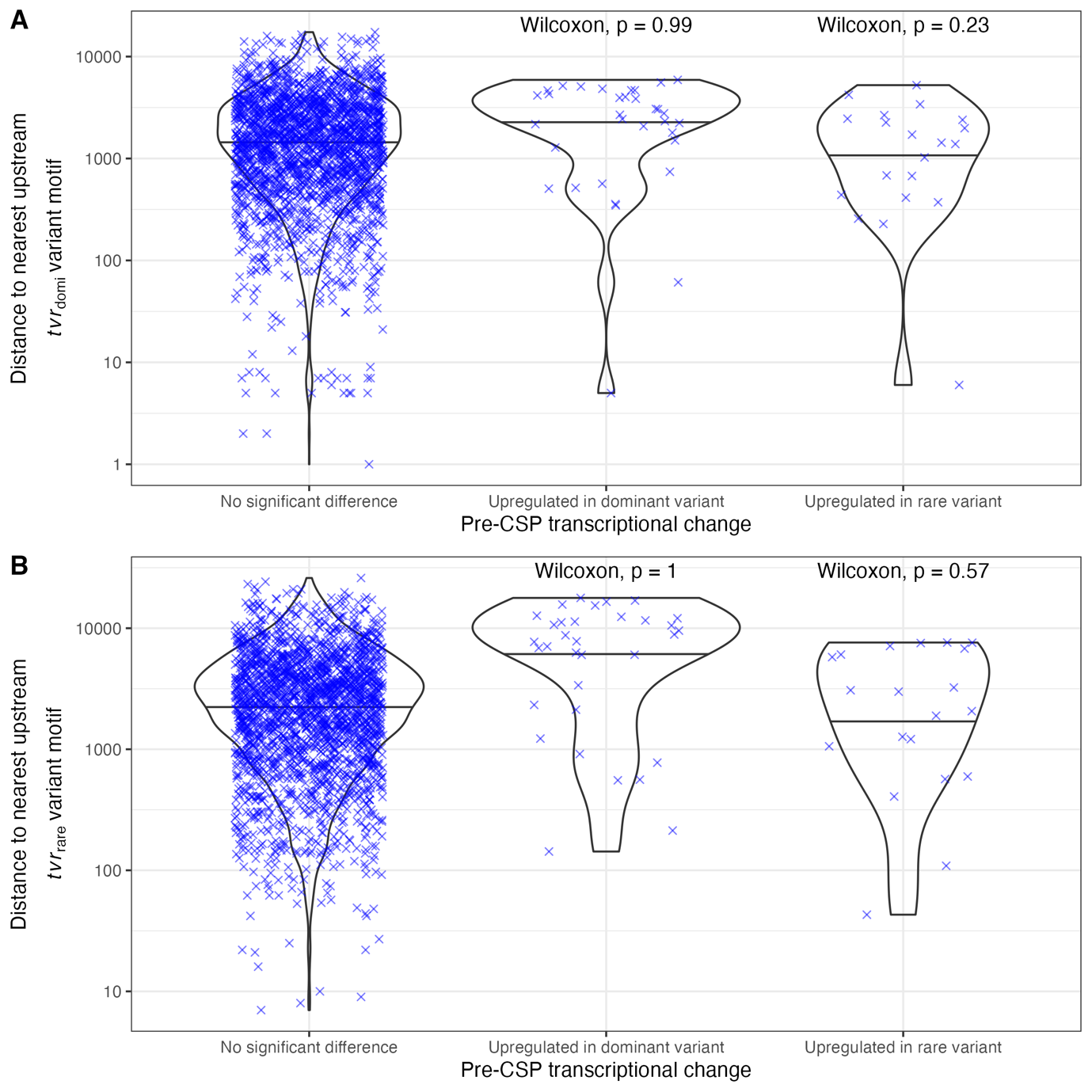


**Figure S16** Violin plots showing the relationship between *Spn*IV methylation sites and transcriptional variation. Each point represents the distance between a protein coding sequence’s translational start site, and the nearest upstream *Spn*IV methylation site based on (A) *tvr*_domi_ motifs and (B) *tvr*_rare_ motifs. Coding sequences (CDSs) were classified based on whether they exhibited a significant difference in transcription between RMV7 *tvr*_domi_::Janus and RMV7 *tvr*_rare_::Janus prior to the addition of CSP. A one-tailed Wilcoxon rank sum test was used to test the hypothesis that CDSs that differed in expression significantly between the two phase variants pre-CSP would be closer to upstream variable methylation sites than the equivalent distances for CDSs that were not associated with strong changes in transcription. No evidence was found of differential expression between the variants being associated with greater proximity of *Spn*IV methylation sites to a coding sequence’s start codon.

**A**

**B**

**C**

**D**

**Figure S17** Spatial relationship between methylation sites and differentially expressed genes. Four genes that significantly differed in levels of transcription between the pre-CSP RMV7 *tvr*_domi_::Janus and RMV7 *tvr*_rare_::Janus samples had *Spn*IV methylation sites within 100 nt of their start codon (Fig. S16). In three cases, this corresponded to *tvr*_domi_ methylation sites shortly upstream of genes within operons: (A) IONPJBJN_00030, which is separated from the upstream gene by only six bases; (B) IONPJBJN_00519, which is within PRCI*_dnaN_*; and (C) IONPJBJN_01032, which is the final gene (*csbD*) in an operon that appears to be part of the MgrA regulon (Fig. S21). Therefore these DNA modifications are unlikely to directly affect the initiation of transcription of the displayed loci, as they are distant from the typical sites of transcriptional regulation, near the relevant promoters. (D) A single gene, *piuA* (IONPJBJN_02012), is the first in an operon, and has a *tvr*_rare_ motif within its putative promoter sequence. This operon is upregulated in RMV7_rare_, and this may represent a direct link between methylation and changes in gene expression.

**Figure S18** Violin plot comparing the transformation efficiency of RMV_rare_ and a mutant in which *piuA* had been disrupted by a Janus cassette. Each individual point represents an independent transformation experiment. The horizontal line within the violins shows the median for each genotype. The disruption of *piuA* did not detectably reduce the transformability of RMV7_rare_, suggesting the methylation site within its promoter and the associated upregulation in RMV7_rare_ did not cause the observed phenotypic differences between the variants.


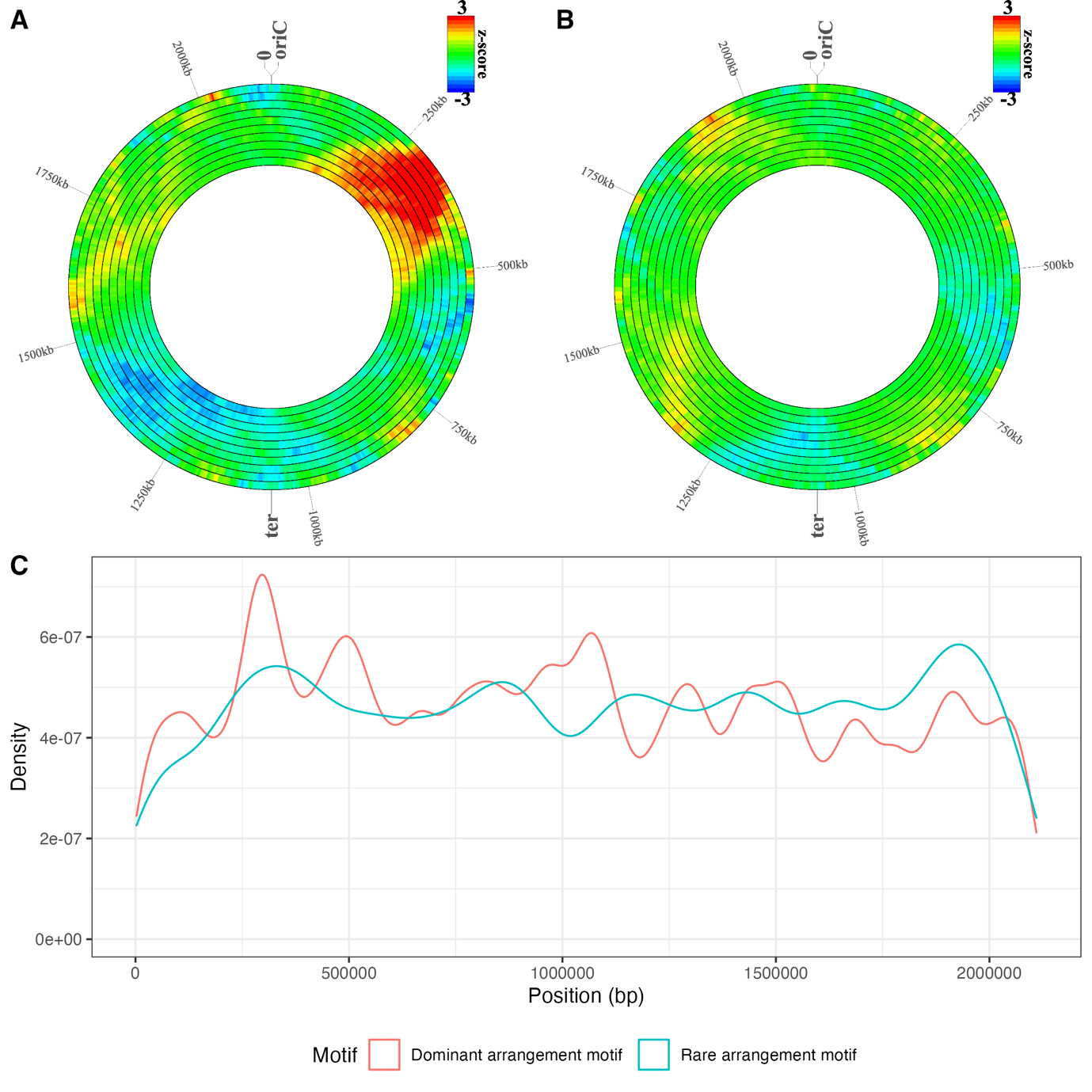


0

0

**Figure S19** Distribution of methylation motifs across the chromosome. The distribution of the (A) *tvr*_domi_ (TGAN_7_TCC) and (B) *tvr*_rare_ (TGAN_7_TATC) motifs across both strands of the RMV7_domi_ genome were analysed with DistAMo. The ten rings represent different sizes of sliding windows, increasing from 50 kb in the outermost ring, to 500 kb in the innermost ring, in 50 kb increments. The density of motifs is quantified as a z-score, as indicated by the key. These values are shown as a heatmap across the chromosome of RMV_domi_. The red regions correspond to a local over-representation of motifs, whereas the blue regions correspond to a local under-representation. (C) Line graph comparing the density of *tvr*_domi_ and *tvr*_rare_ motifs across the RMV7_domi_ genome.


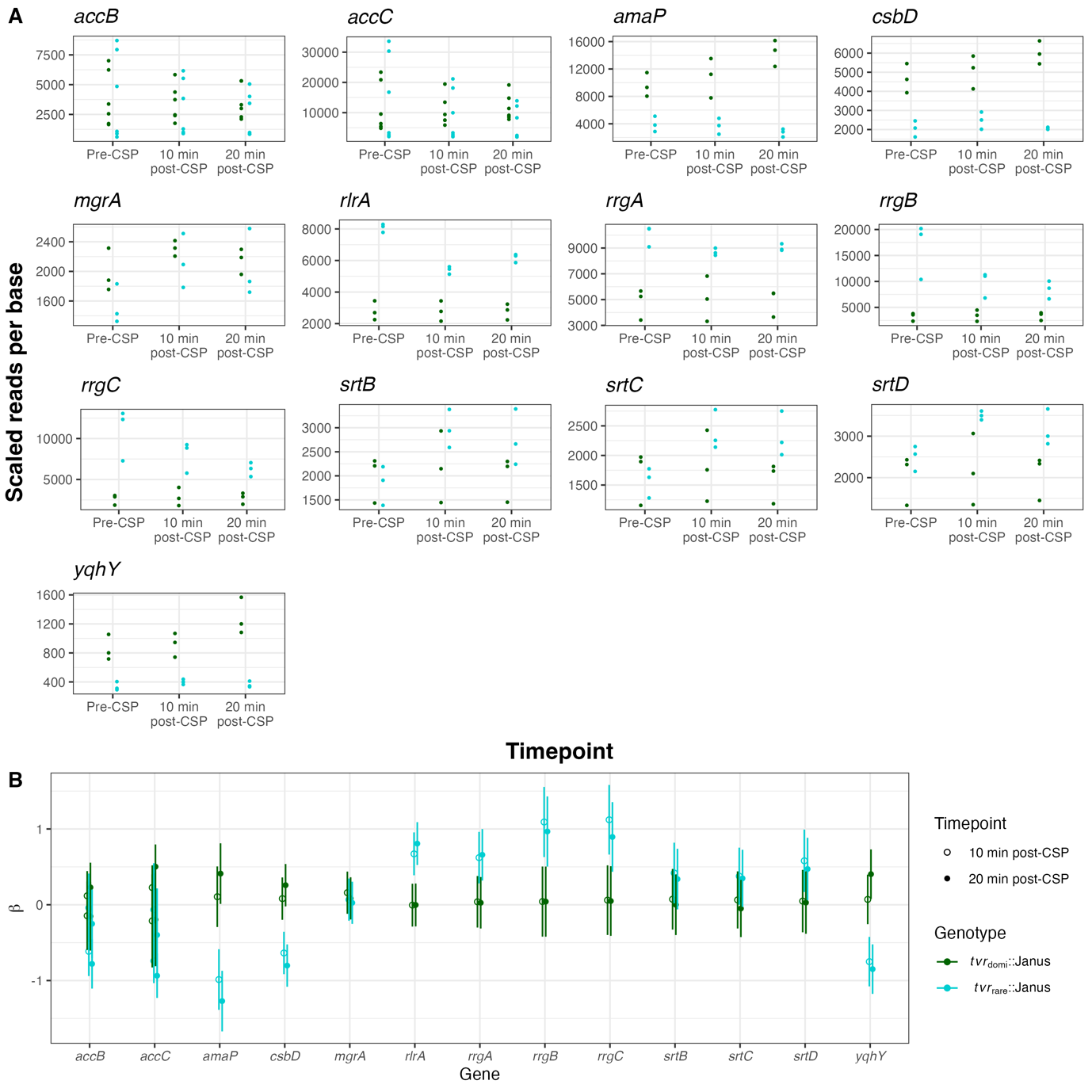


**Figure S20** Quantification of the expression of *mgrA* regulon genes using RNA-seq data. Data are displayed as in Fig. S11.


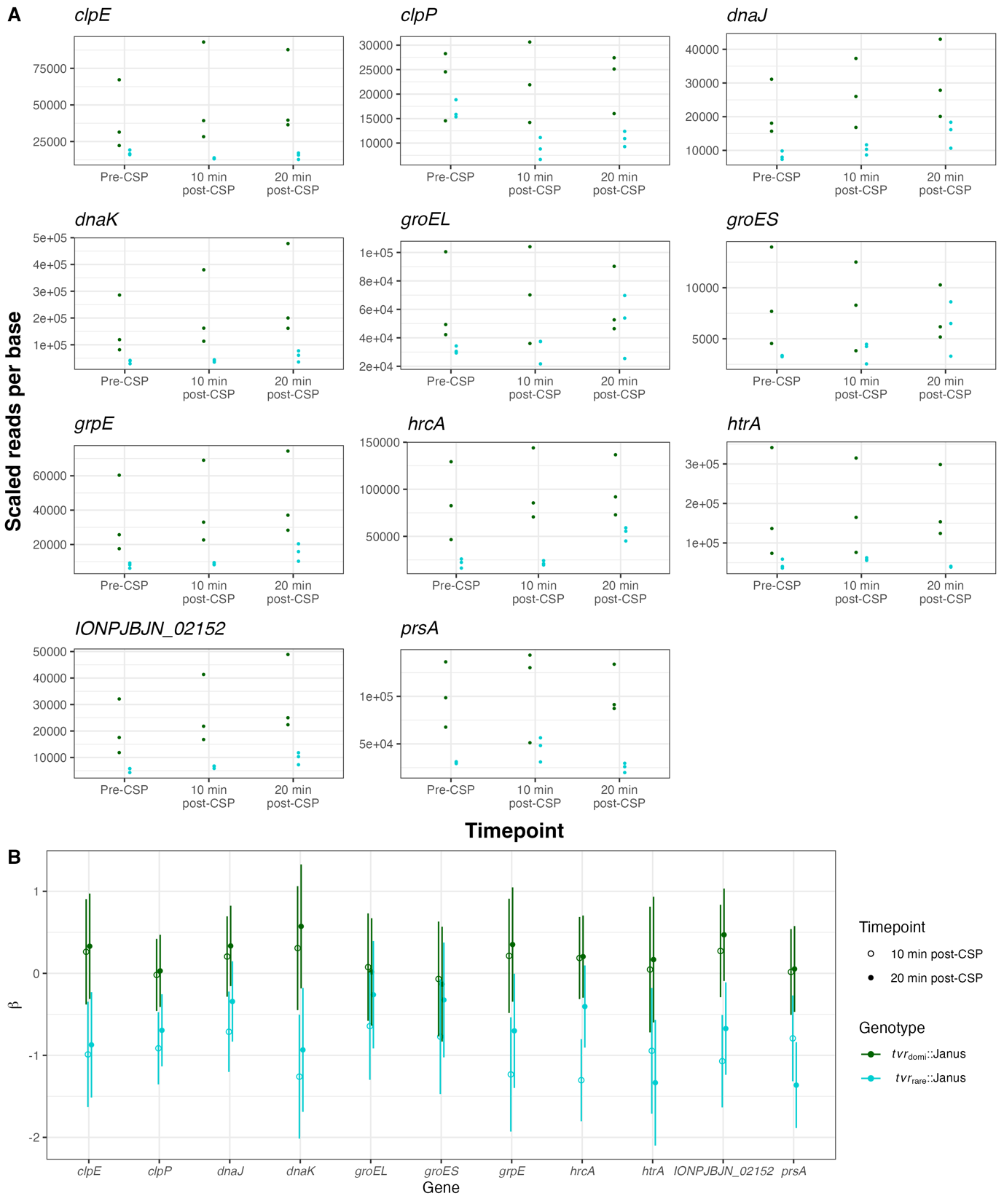


**Figure S21** Quantification of the expression of stress response and chaperone genes using RNA-seq data. Data are displayed as in Fig. S11.


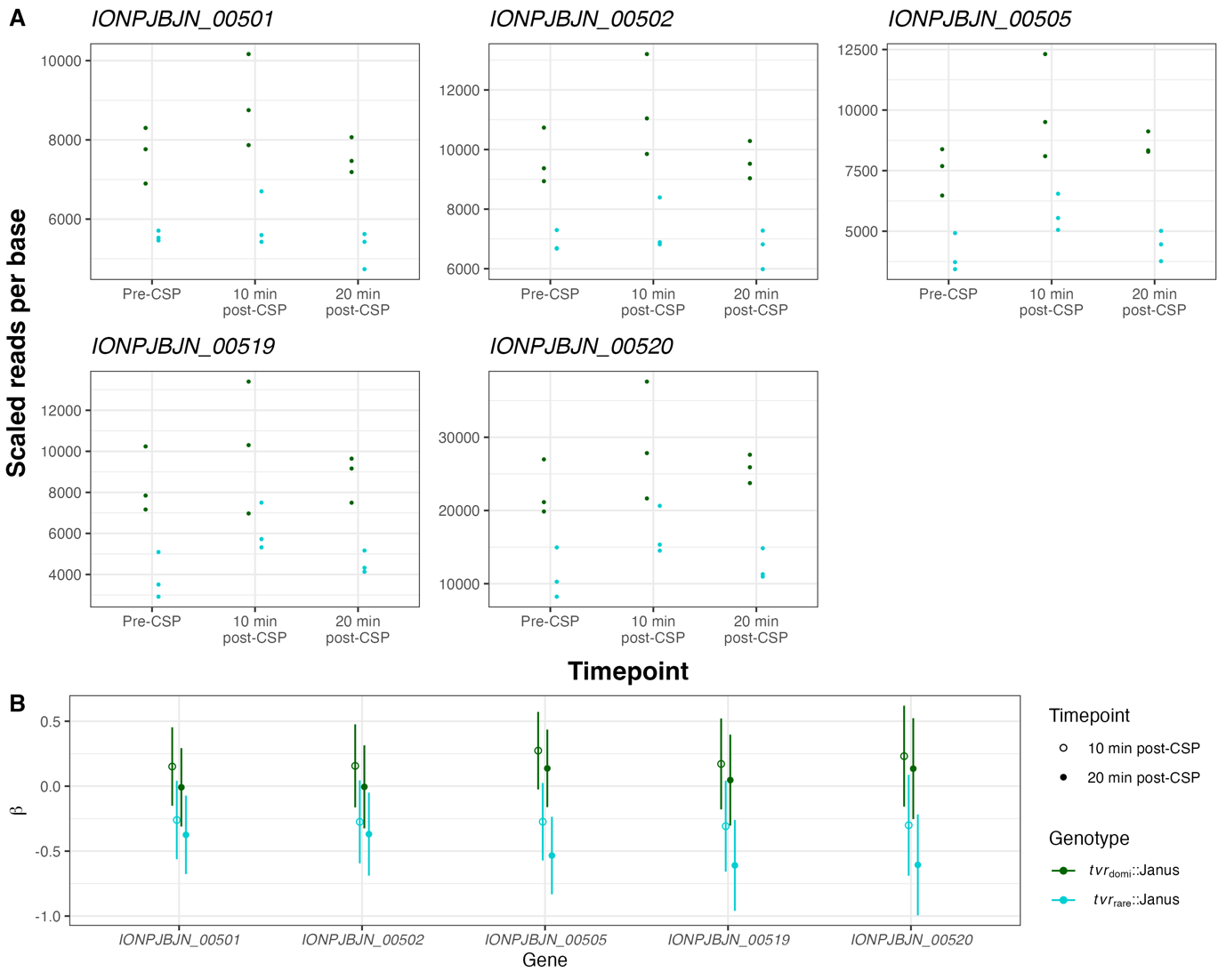


**Figure S22** Quantification of the expression of large PRCI*_dnaN_* genes using RNA-seq data. These were all more active in RMV7 *tvr*_domi_::Janus. Data are displayed as in Fig. S11.


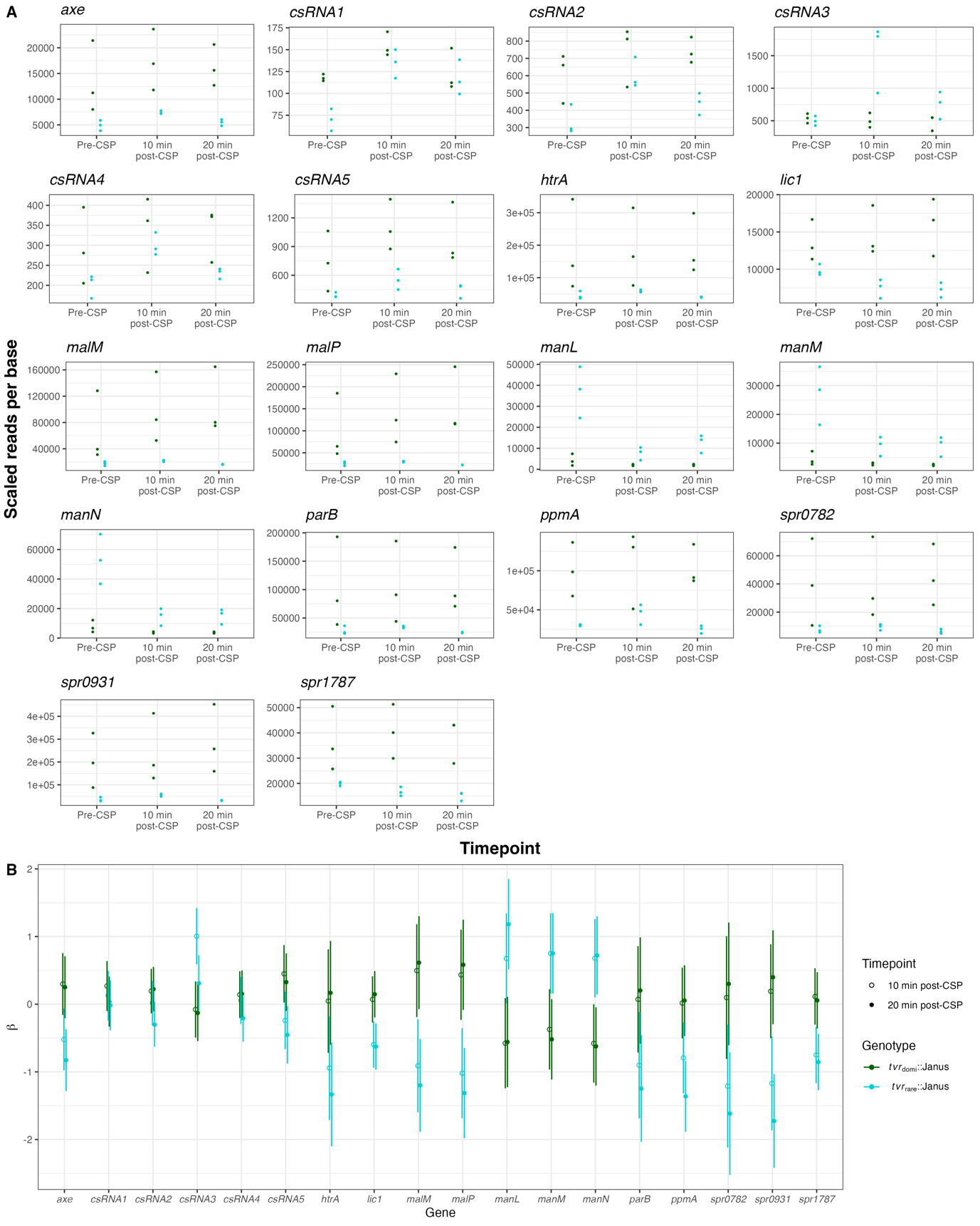


**Figure S23** Quantification of the expression of *ciaRH* regulon genes using RNA-seq data. Data are displayed as in Fig. S11.

**Figure S24** Violin plot comparing the transformation efficiency of mutants in which *ciaRH* or *htrA* were disrupted in the RMV7_wt_ and RMV7_rare_ backgrounds. Each individual point represents an independent transformation experiment. The horizontal line within the violins shows the median for each genotype. Disruption of *ciaRH* had limited impact on the transformation efficiency of either genotype. However, disruption of *htrA* increased the transformation efficiency of the already highly transformable RMV7_rare_.

**Figure S25** Violin plot comparing the transformation efficiencies of RMV7_rare_ mutants in *manLMN*, *tfoX* and *yjbK* with the parental genotype. The data shown are the same as displayed for the experiments conducted in unsupplemented media in Fig. 3A, but here the two-tailed Wilcoxon rank sum test compares the parental genotype with the mutants. Significance between results is coded as: *p* < 0.05, *; *p* < 0.01, **; *p* < 10^-3^, ***; *p* < 10^-4^, ****.

**Figure S26** Effects of ManLMN and carbon source on *in vitro* growth of RMV7_wt_ and RMV7_rare_. Both RMV7_wt_ and RMV7_rare_, and mutants of each lacking *manLMN*, were grown in unsupplemented mixed media liquid (a nutrient-rich mix of Todd-Hewitt media, with 0.5% yeast extract, and Brain-Heart Infusion). This was either unsupplemented, or supplemented with one of six additional carbon sources, indicated by the colour of the growth curve. Growth was measured using the optical density at 600 nm (OD_600_) across three biological replicates. The lines represent the median optical density, and the shaded ribbon shows the range of the replicates. There was little evidence of the loss of ManLMN causing a substantial decrease in growth. The only carbon source found to increase the final population size was *N*-acetylglucosamine in RMV7_wt_, which was not observed when *manLMN* was disrupted in this background


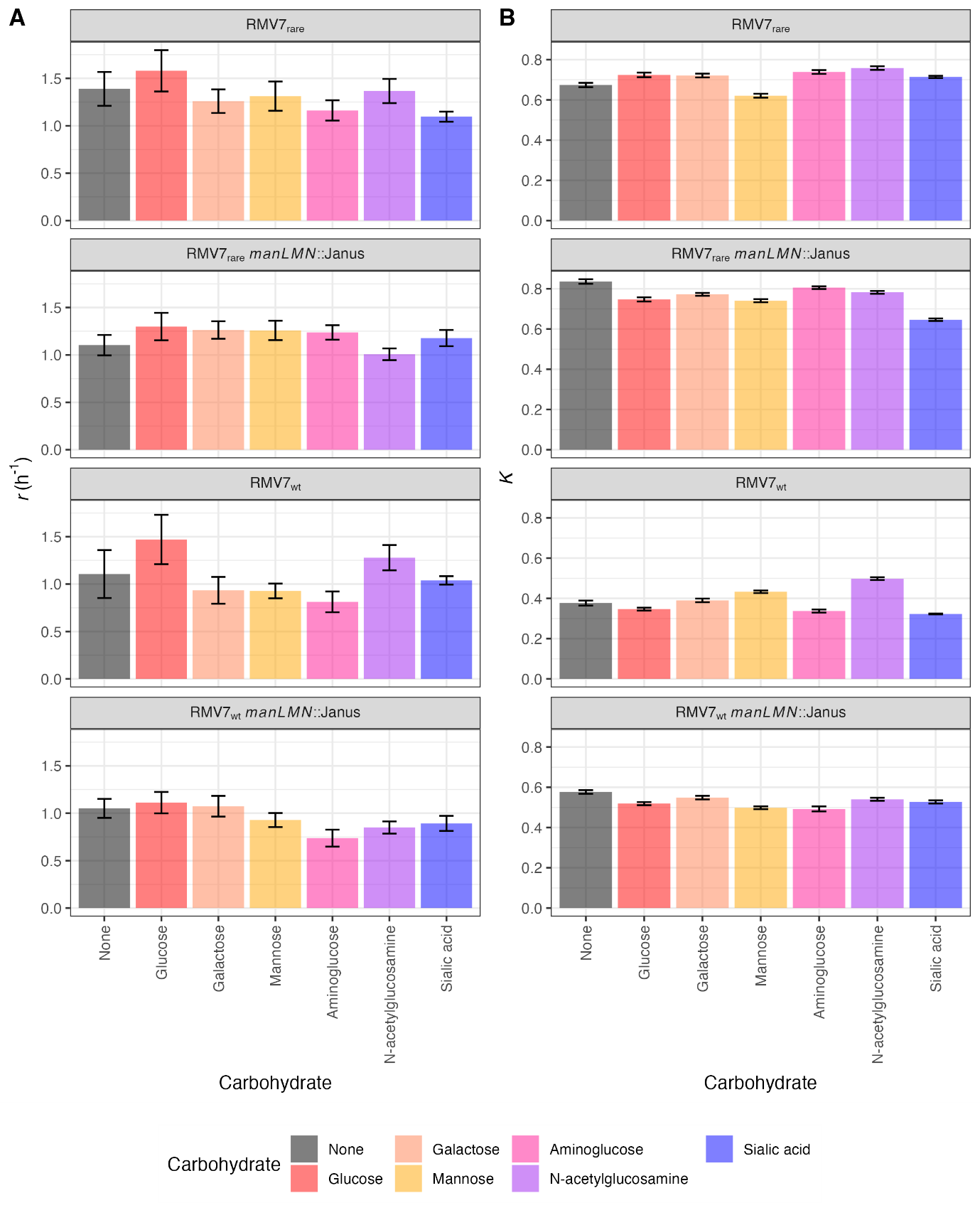


**Figure S27** Barplots summarising the effects of ManLMN and carbon source supplements on growth of RMV7_domi_ and RMV7_rare_. The plots are separated by variant and whether *manLMN* was disrupted by a Janus cassette insertion. The colours of the bars show the carbon source supplement in the media. The height of the bars show the estimates of logistic model parameters, and the error bars show the 95% confidence intervals of these estimates. (A) Estimates of the reproduction rate, *r*. (B) Estimates of the carrying capacity, *K*.

**Figure S28** Confirming the role of *manL* in regulating the competence system. The large size of the *manLMN* operon, combined with the low transformation efficiency of the *manLMN*::Janus genotype, made it impractical to restore the entire operon. Hence a second mutant was constructed in which only *manL* was disrupted with a Janus cassette. This smaller mutation could then be reversed in the more transformable RMV7_rare_ cells. The data are shown as in Fig. 3A. This shows disruption of *manL* reduced the transformation efficiency of RMV7_rare_ to a similar extent as disruption of the entire *manLMN* operon. Restoration of the *manL* gene resulted in a more transformable genotype that responded to GlcNAc, confirming this locus was responsible for the decrease in transformation efficiency, and lack of response to GlcNAc, in the *manL*::Janus mutant.

**Figure S29** Location of the *tfoX* gene in the *S. pneumoniae* chromosome. Blue arrows represent intact genes, and brown arrows represent gene fragments. The *tfoX* gene in RMV7 is found at a conserved position, shortly upstream of the *comEC* and *comEA* genes. These genes encode the main DNA entry pore of the competence machinery. The intervening gene fragments are part of a pseudogene predicted to have encoded an alanine dehydrogenase.
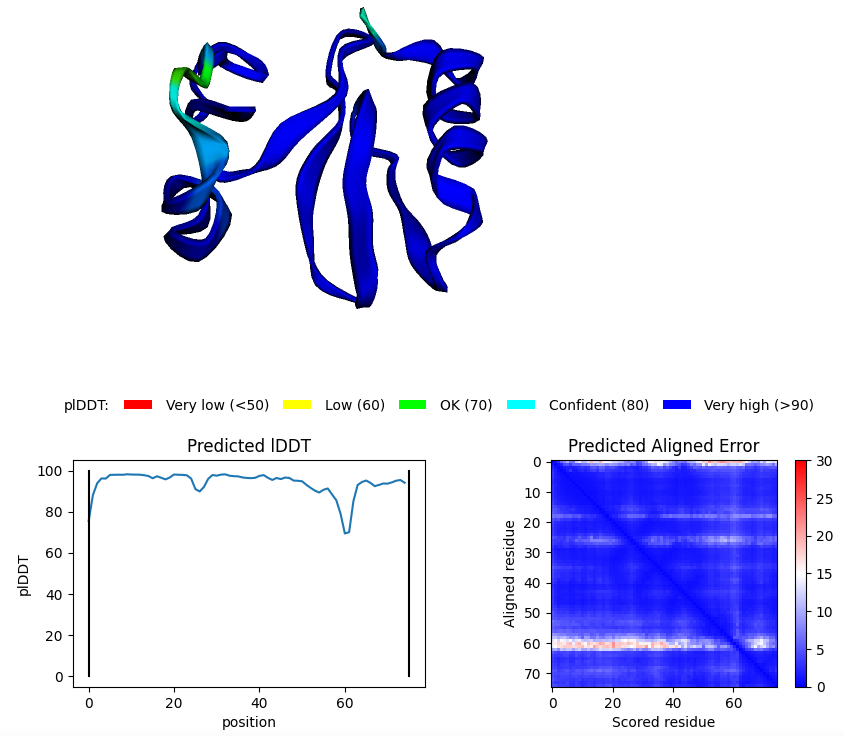
**Figure S30** Predicted structure of TfoX*_Spn_*. (A) The amino acid sequence of RMV7_domi_ *tfoX* (IONPJBJN_02097) was analysed using the AlphaFold2 pipeline with default settings. The protein structure prediction at the top shows a four-strand beta sheet flanked by alpha helices. The structure is coloured according to the confidence of the prediction, as quantified by the per-residue local distance difference test (plDDT). (B) A line graph of plDDT over the length of the protein. This shows the algorithm has high confidence in the predicted structure. (C) A heatmap showing the uncertainty from the alignment. This shows there to be few regions of uncertainty in the prediction.

**A**

**C**

**B**


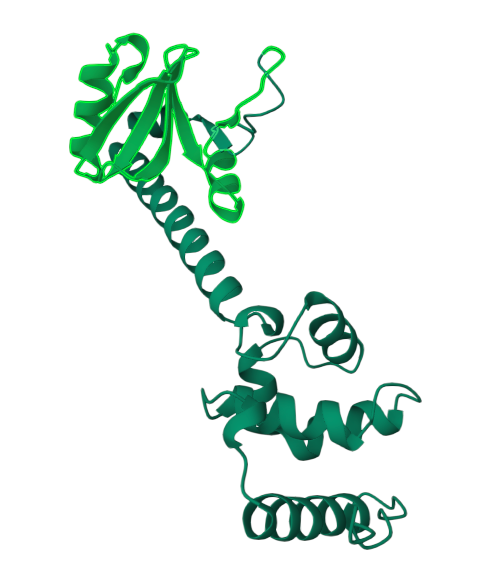

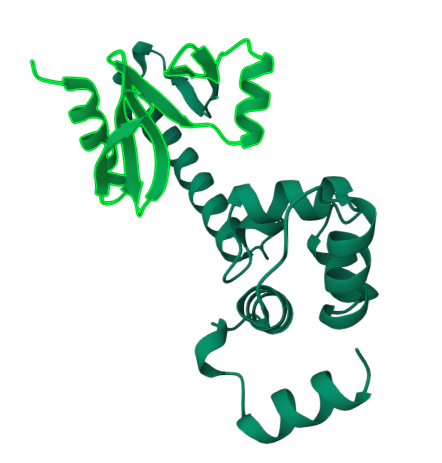

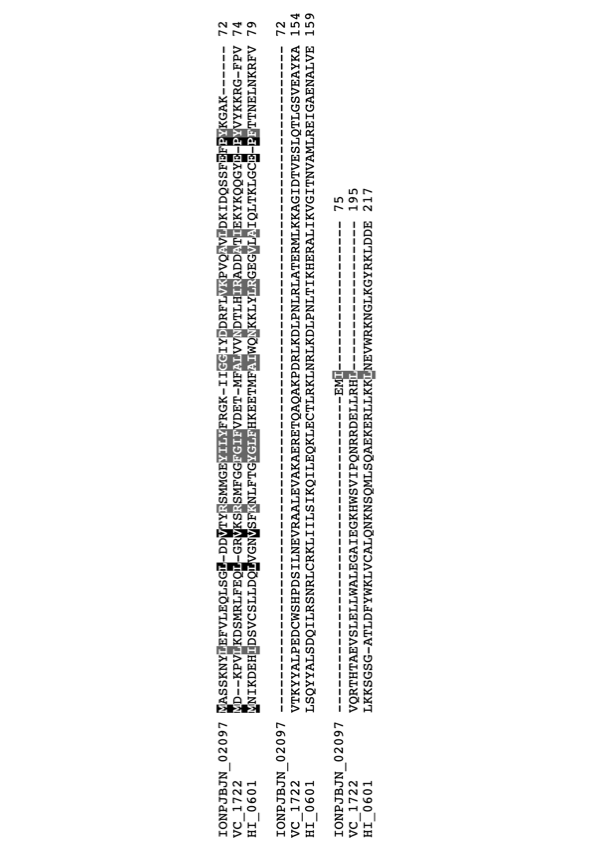


**B**

**C**

**A**

**Figure S31** (A) Alignment of the TfoX proteins of *Vibrio cholerae* (VC_1772 from *V. cholerae* ATCC 39315) and *Haemophilus influenzae* (HI_0601 from *H. influenzae* Rd) with the orthologue in RMV7, IONPJBJN_02097. Columns are coloured black where an amino acid is conserved at a position, and grey when amino acids are similar at a position. (B) The predicted structure of HI_0601, inferred using AlphaFold (AlphaFoldDB identifier AF-P43779-F1). The first 72 amino acids are highlighted in light green. These have a similar structure to that predicted for TfoX*_Spn_* (Fig. S30): two alpha helices linked by a four-strand beta sheet. (C) The predicted structure of VC_1772, inferred using AlphaFold (AlphaFoldDB identifier AF-Q9KRC0-F1). The first 72 amino acids are highlighted in light green. These have a similar structure to that predicted for TfoX*_Spn_* and the N terminal region of HI_0601.


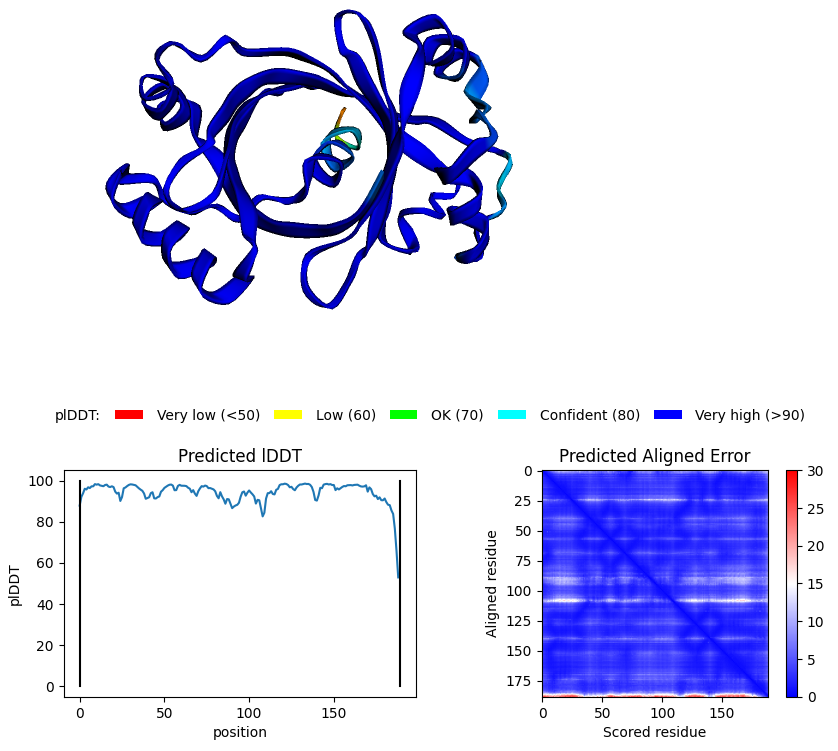


**A**

**B**

**C**

**Figure S32** Predicted structure of YjbK*_Spn_*. (A) The amino acid sequence of RMV7_domi_ *yjbK* (IONPJBJN_01639) was analysed using the AlphaFold2 pipeline with default settings. The protein structure prediction at the top shows the barrel, composed of eight beta strands, that is characteristic of CYTH proteins. The structure is coloured according to pIDDT (see Fig. S30). (B) A line graph of pIDDT over the length of the protein. This shows the algorithm has high confidence in the predicted structure. (C) A heatmap showing the uncertainty from the alignment. This shows the only region of uncertainty in the structure is at the very C terminus.


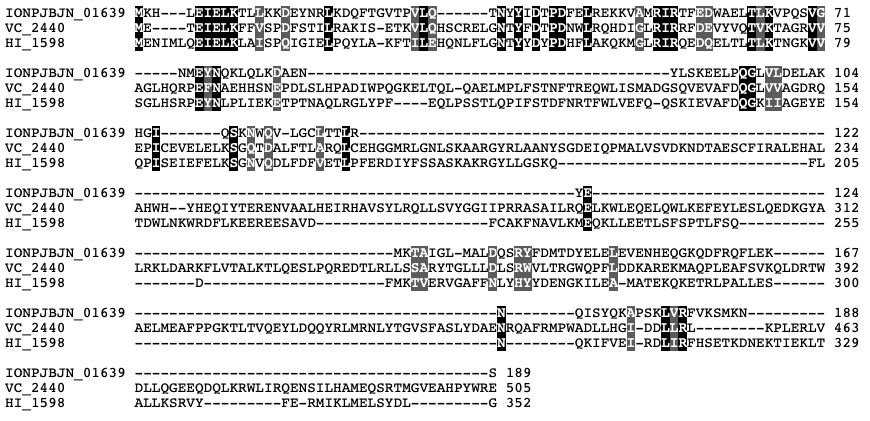


**B**

**C**

**A**


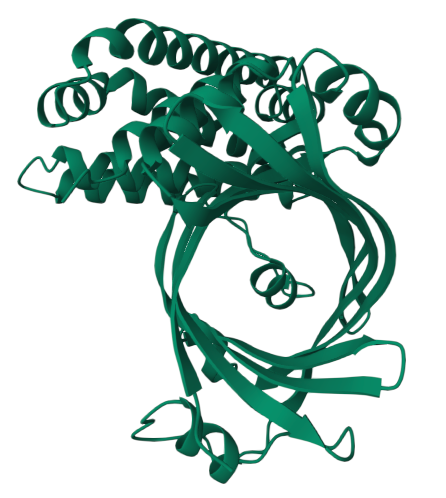

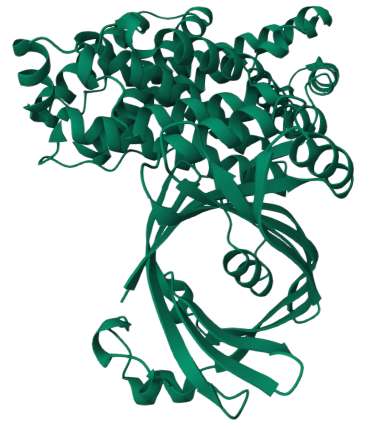
**Figure S33** (A) Alignment of CyaB adenylate cyclase proteins of *Vibrio cholerae* (VC_2440 from *V. cholerae* ATCC 39315) and *Haemophilus influenzae* (HI_1598 from *H. influenzae* Rd) with YjbK of RMV7, IONPJBJN_01639. Columns are coloured black where an amino acid is conserved at a position, and grey when amino acids are similar at a position. (B) The predicted structure of HI_1598, inferred using AlphaFold (AlphaFoldDB identifier AF-P45267-F1). This structure has a beta barrel structure, like YjbK. (C) The predicted structure of VC_2440, inferred using AlphaFold (AlphaFoldDB identifier AF-Q9KPD2-F1). This structure has a beta barrel structure, like YjbK.

**
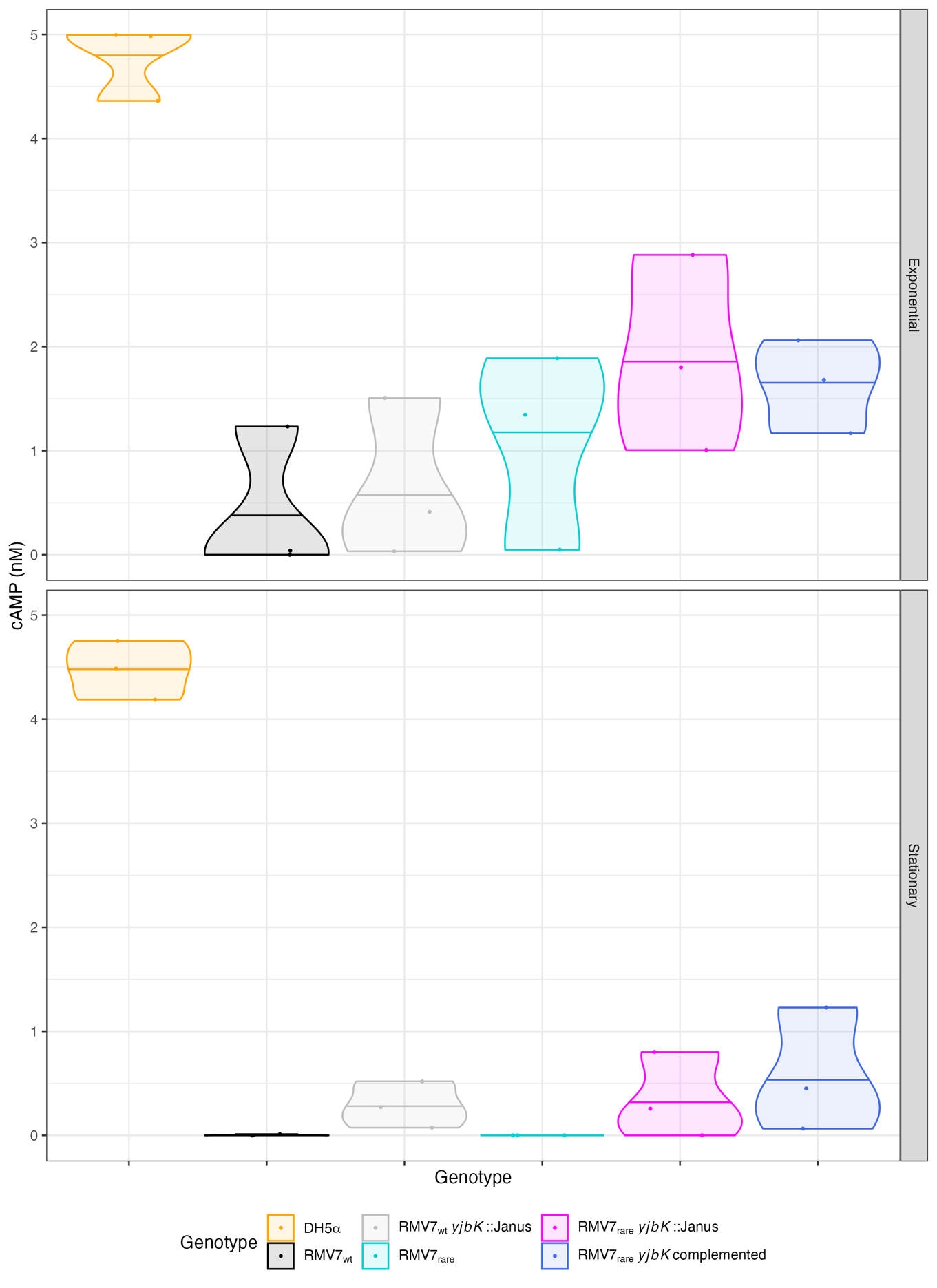
**

**Figure S34** Violin plots showing the concentration of 3’,5’-cAMP in samples taken from exponential and stationary phase cultures of *E. coli* DH5⍺, and *S. pneumoniae* RMV7_wt­_ and RMV7_rare_ genotypes differing in whether *yjbK* was intact or not. The colours indicate the genotypes from which the samples were extracted.

**Figure S35** Effect of exogenous 3’,5’-cAMP on *S. pneumoniae* transformation efficiency. The RMV7_wt_, RMV7_domi_ and RMV7_rare_ genotypes were transformed in liquid media with and without a supplement of 4 mM 3’,5’-cAMP. This addition did not have any detectable effect on the transformation efficiency of any of the genotypes.

**Figure S36** Analysing GlcNAc metabolism and signalling through comparing growth curves of RMV7_rare_ *nagA*::Janus, *tfoX*::Janus and *yjbK*::Janus mutants. The line graph summarises three replicate growth experiments undertaken using mixed media. The solid line shows the median recorded optical density, and the shaded area shows the range of observations.


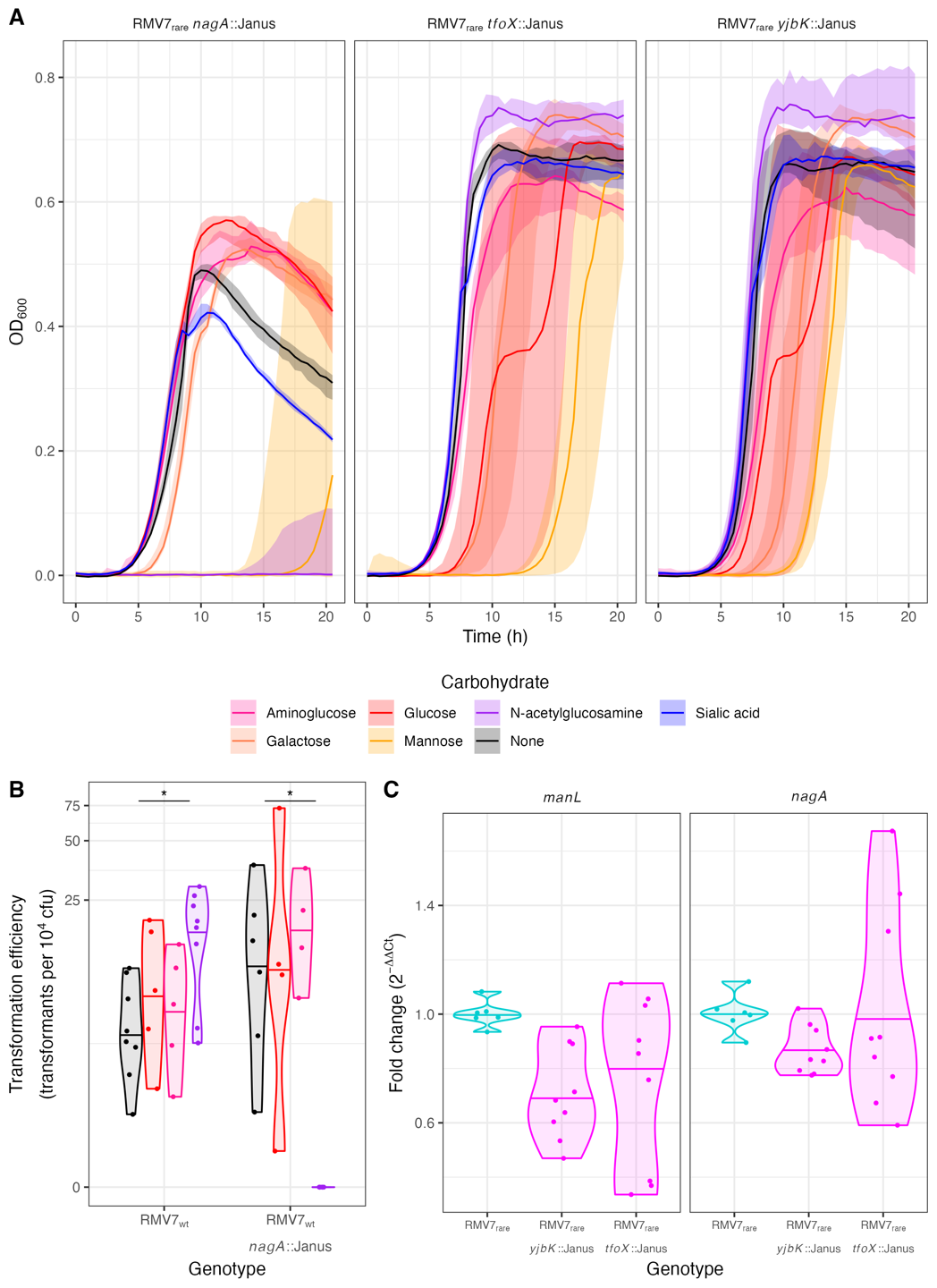


**Figure S37** Analysing GlcNAc metabolism and signalling through comparison of *nagA*::Janus, *tfoX*::Janus and *yjbK*::Janus mutants. (A) Growth curves of RMV7_rare_ mutants in mixed media supplemented with different carbon sources. The *nagA*::Janus mutant exhibited a strong growth defect in the presence of GlcNAc. This is consistent with NagA being the primary metabolic enzyme processing the cytotoxic compound GlcNAc-6-phosphate. The *tfoX*::Janus and *yjbK*::Janus mutants both grew to elevated densities in the presence of a GlcNAc supplement. (B) Transformation efficiency of RMV7_rare_ *nagA*::Janus in presence of four different carbon source supplements. Consistent with the effects on growth in panel (A), no transformed colonies were recovered from the *nagA*::Janus mutant in the presence of a GlcNAc supplement. (C) Transcription of *manL* and *nagA* were measured in RMV7_rare_, RMV7_rare_ *tfoX*::Janus and RMV7_rare_ *yjbK*::Janus grown to early exponential phase (OD_600_ = 0.2). The expression of *nagA*, affecting metabolism of GlcNAc, was not affected by disruption of *tfoX* or *yjbK*. The transcription of *manL* was reduced by both mutations, suggesting the effects of *tfoX* and *yjbK* could be mediated through changing the level of the ManLMN transporter within cells.

**Figure S38** Replication of the link between metabolism and transformation efficiency in the laboratory isolate *S. pneumoniae* R6. The *manLMN*, *tfoX* and *yjbK* genes were each disrupted with a Janus cassette in R6. Transformation efficiencies were measured with the bacteria grown in a nutrient-poor chemically-defined medium (see Methods). All three mutants had reduced transformation efficiencies. These differences were not observed when R6 was grown in rich media.


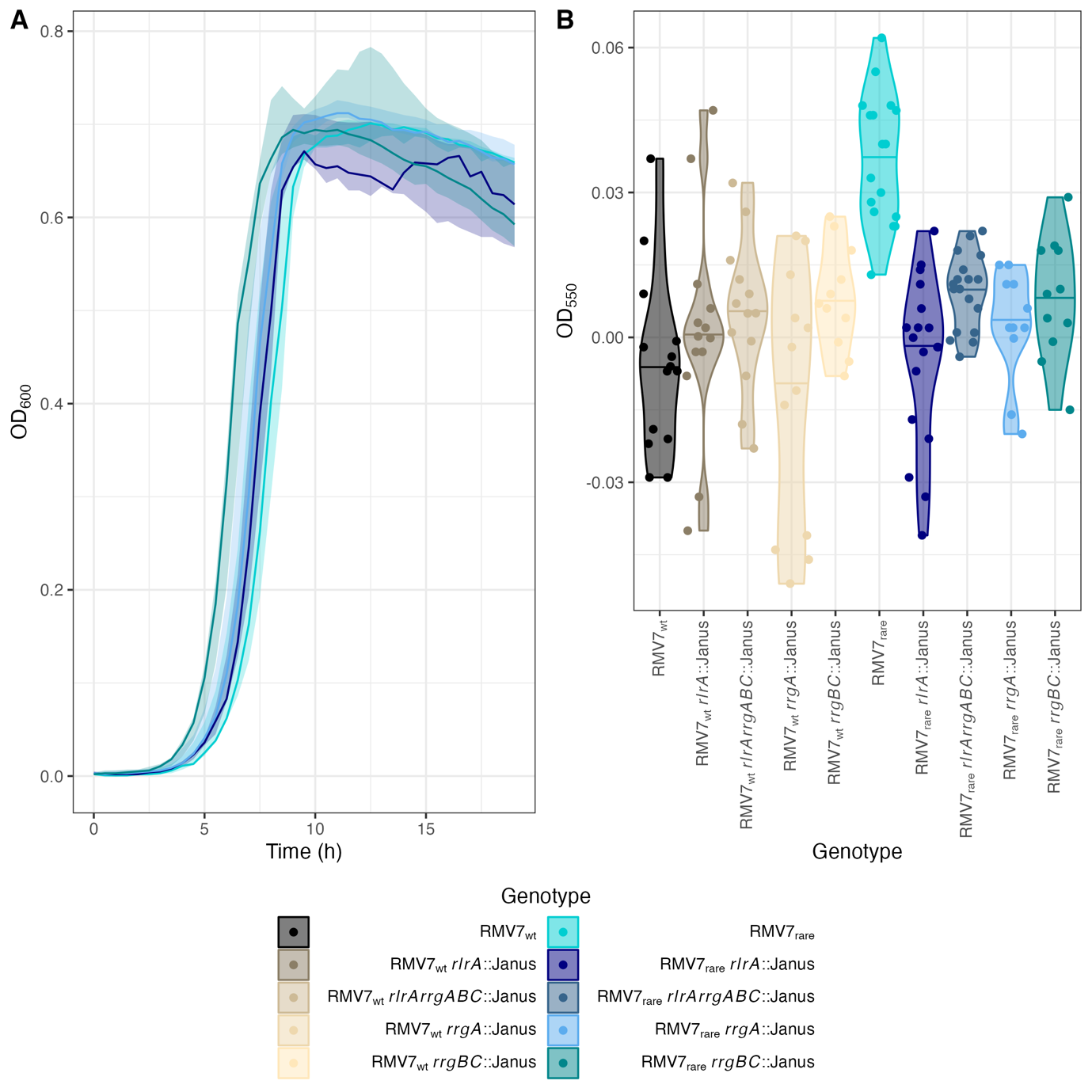


**Figure S39** Effect of type 1 pilus expression on pneumococcal phenotypes. (A) Growth curves of RMV7_rare_ and mutants in which the genes for the type 1 pilus regulator (*rlrA*) or structural proteins (*rrgABC*) were disrupted using a Janus cassette. The median (solid line) and range (shaded area) of observations were calculated from three replicates. The loss of the pilus genes has little effect on growth. (B) Biofilm formation of RMV7 genotypes and mutant derivatives lacking type 1 pili. The greatest adherence of cells to the abiotic surface was observed with the RMV7_rare_ variant, which expressed the type 1 pilus more strongly than the RMV7_wt_ variant. Correspondingly, loss of the regulatory or structural pilus genes had little effect on biofilm formation in RMV7_wt_, but substantially decreased biofilm formation by RMV7_rare_.


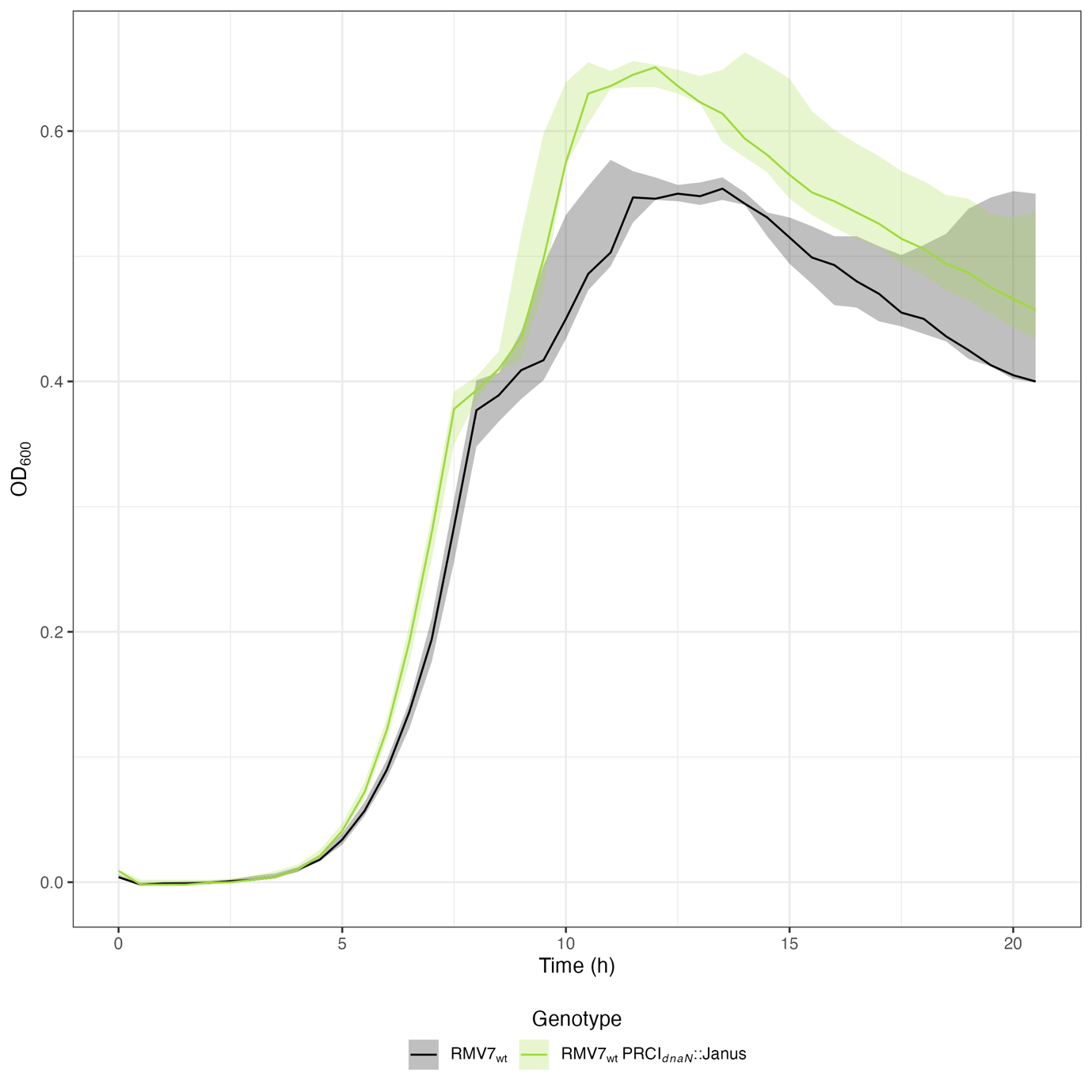


**Figure S40** Growth curves of RMV7_wt_ and RMV7_wt_ PRCI*_dnaN_*::Janus in mixed media. In each plot, the lines represent the median OD_600_, and the shaded ribbon shows the range of three replicates. This demonstrates that the deletion of PRCI*_dnaN_* increases the growth rate, and carrying capacity, of the RMV7_wt_, suggesting the activity of this element causes intracellular stress.


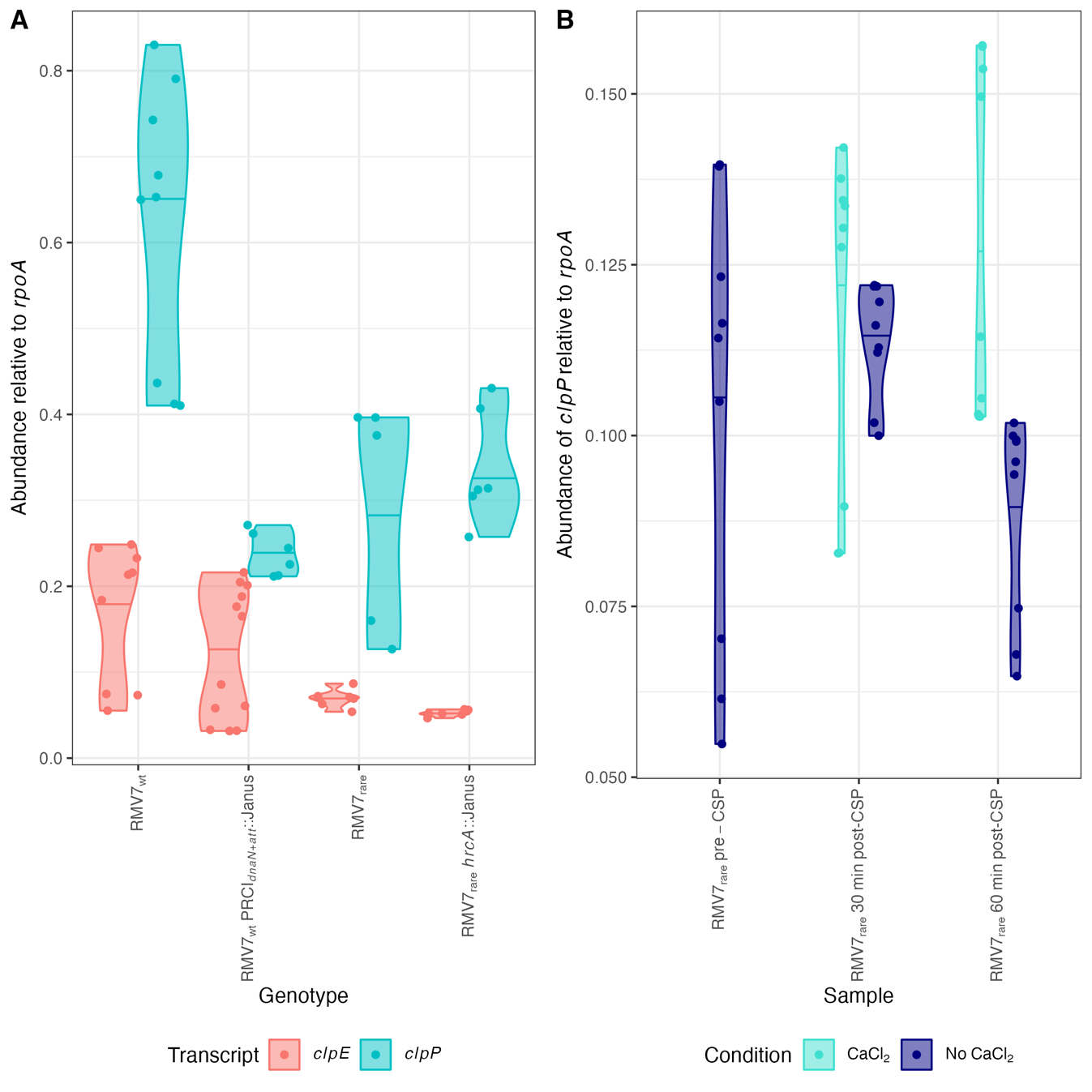


**Figure S41** Expression of *clpE* and *clpP*. In these plots, each point represents one of the three technical replicate measurements of each biological replicate. (A) Violin plot showing the relative abundance of *clpE* and *clpP* transcripts in different genotypes. The *clpP* gene was most highly transcribed in RMV7_wt_, in which the PRCI is highly active. The absolute expression of *clpP*, and the expression relative to *clpE*, was reduced when the PRCI was removed from RMV7_wt_, suggesting expression was induced by the stress of the element’s activity (data also shown in Fig. 4C). Correspondingly, *clpP* expression was similarly low in RMV7_rare_, in which the PRCI is less active. The deletion of *hrcA* had relatively little effect on the expression of *clpE* or *clpP* in the absence of competence induction. (B) Violin plot showing the effect of CSP and Ca^2+^ on *clpP* expression in RMV7_rare_. In the absence of a Ca^2+^ supplement, relatively little change in *clpP* expression was observed after the induction of competence. The addition of a Ca^2+^ supplement elevated expression of *clpP*. This likely reflected the increased suppression of other chaperones, due to the increased binding of HrcA to CIRCE motifs. Hence although ClpP does not appear to be part of the direct HrcA regulon (panel A), it is likely to be indirectly affected by the overlap with the functional roles of other chaperone proteins within the cell following the induction of competence.

**Figure S42** Growth curves of RMV7_wt_ and RMV7_rare_ chaperone mutants in mixed media. In each plot, the lines represent the median OD_600_, and the shaded ribbon shows the range of three replicates. The growth of RMV7_wt_ genotypes is shown on the left, with RMV7_rare_ genotypes on the right. The top row shows the growth of cells under standard culturing conditions, whereas the bottom row shows the growth of pneumococci at 40 °C. These “heat shock” assays required first growing the cells at 35 °C to an OD_600_ of 0.2, to establish replication, before then being grown at 40 °C over a further 20 hours.

**Figure S43** Violin plots showing gene expression, as quantified by qRT-PCR, in RMV7_rare_ and a mutant derivative in which the *hrcA* chaperone regulator gene was disrupted by a Janus cassette. The points for each gene correspond to three technical replicate assays of each biological replicate. The horizontal line on the violin plot shows the median relative abundance for each gene in each genotype. The absence of any change in *ciaR* expression demonstrated the effect on transformation was independent of CiaRH.

**Figure S44** Replication of the HrcA-dependent Ca^2+^-activation of transformation in the laboratory isolate *S. pneumoniae* R6. The *hrcA* gene was disrupted by a Janus cassette, and then restored. Each point represents the transformation efficiency of an individual experiment. The solid lines represent the best fit of a dose-response logistic model. The shaded areas correspond to the 95% confidence intervals. Both of the *hrcA*^+^ genotypes responded to increasing concentrations of Ca^2+^ ions, but the genotype in which *hrcA* was disrupted did not.

**
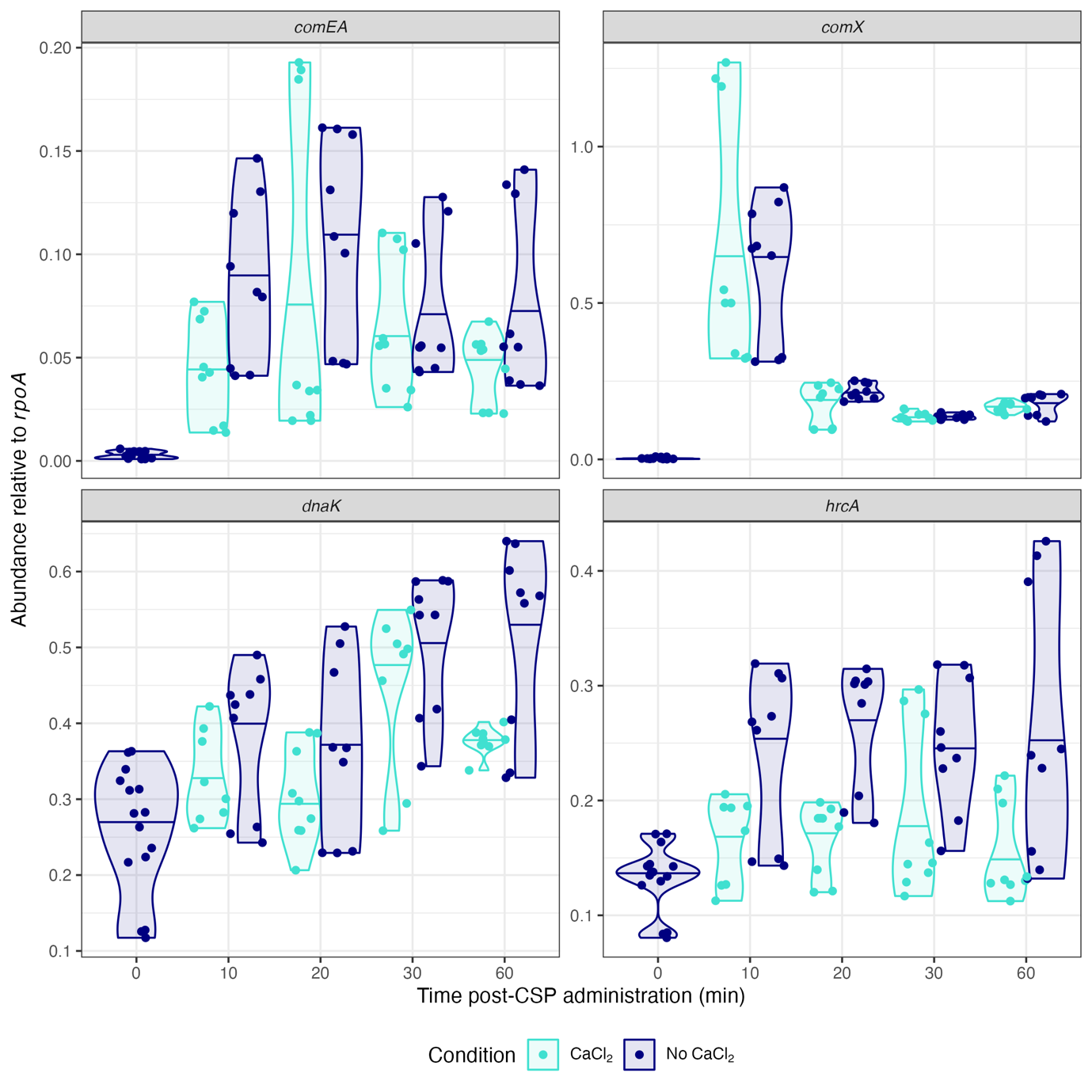
**

**Figure S45** Violin plots showing the effect of Ca^2+^ signalling on the expression of genes regulated by HrcA after the administration of CSP to a culture of RMV7_rare_. Transcription of each gene was quantified relative to *rpoA* by qRT-PCR, using three technical replicate measurements of each of three biological replicates. Ca^2+^ supplementation increases HrcA’s affinity for DNA, and as the protein autorepresses, this decreases *hrcA* transcription. Similarly, *dnaK* is known to be repressed by HrcA. However, in this assay there is only evidence of repression after a 30-60 min delay. By contrast, there was little evidence of Ca^2+^-associated repression of either the early competence gene *comX*, or the late competence gene *comEA*. This suggests HrcA does not broadly repress the competence regulon.**
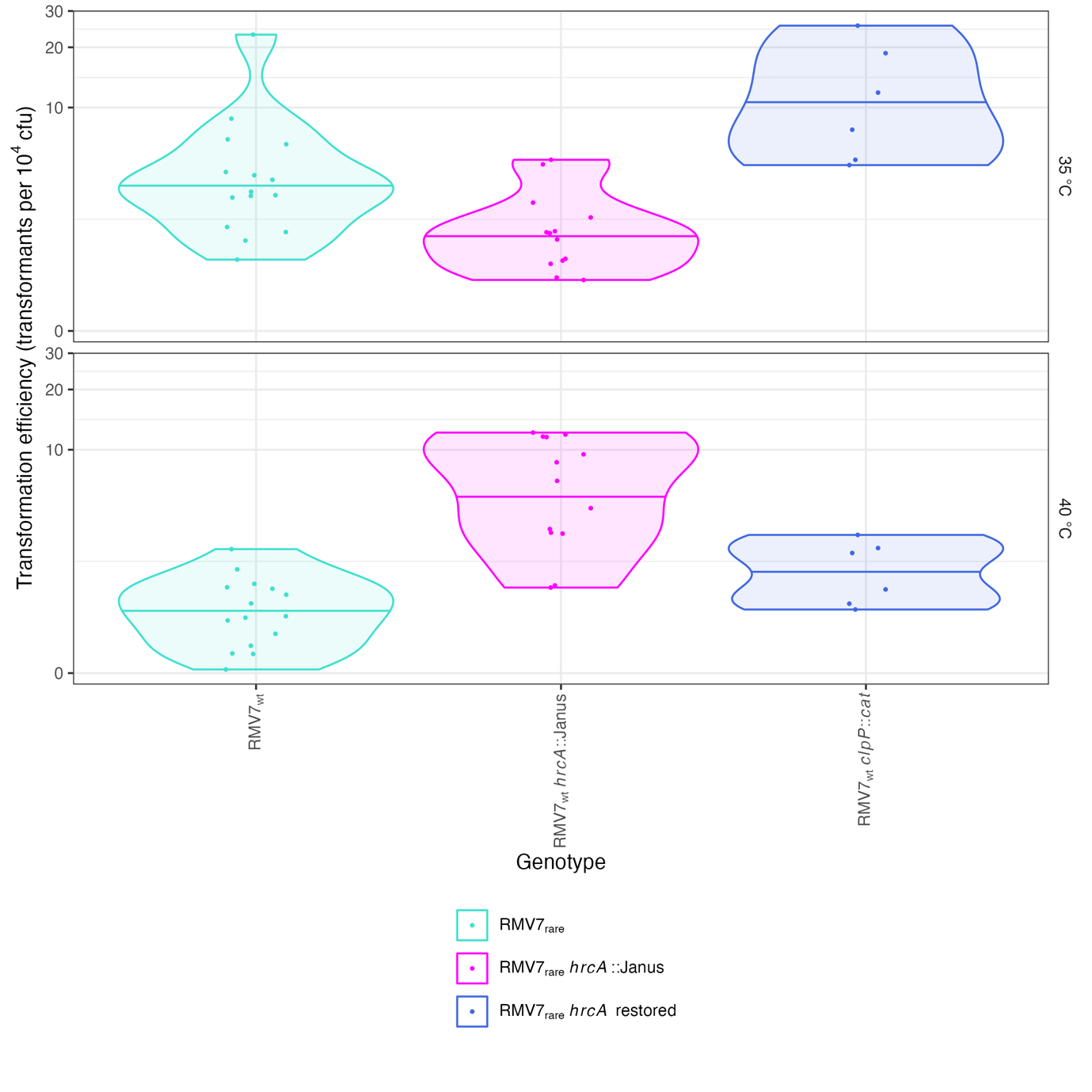
**

**Figure S46** Violin plots showing the transformation efficiency of RMV7_rare_ and mutant derivatives during normal growth (35 °C) or during a 40 °C heat shock. The *hrcA* gene was disrupted in RMV7_rare_ *hrcA*::Janus, and reinstated in RMV7_rare_ *hrcA* restored. Data for RMV7_rare_ and RMV7_rare_ *hrcA*::Janus are reproduced from Fig. 5A. Transformation experiments for RMV7_rare_ *hrcA* restored were conducted separately, but nevertheless show reinstating the *hrcA* gene reverses both the decrease in transformation efficiency at 35 °C, and the increase in transformation efficiency at 40 °C. Each point corresponds to an independent transformation experiment, and the violin plots have a horizontal line indicating the median transformation efficiency of each mutant at each temperature.

**Figure S47** Heterogeneous induction of competence across the pneumococcal population. (A) Four independent replicate experiments on separate cultures of RMV7_rare_ and RMV7_wt_ measured the frequency of transformation with kanamycin and rifampicin resistance markers, each on separate PCR amplicons, in the presence of GlcNAc. Assuming each marker was acquired independently, the expected frequency of double mutants was estimated from the observed frequencies of the single mutants. The observed frequencies of double mutants were higher than those expected for all four replicates with the RMV7_rare_ genotype, and two of the four replicates for the less-transformable RM7_wt_ genotype; in the other two replicates, the expected frequencies of double mutants were much below one cell per experiment, limiting the power to detect a deviation from this number. The excess of observed double mutants, above the expected frequency, is consistent with only a subpopulation of pneumococci within the culture being transformable. (B) Bar plot showing the estimated fraction of pneumococci that are transformable. The ratio of the expected to observed double mutant frequencies is an estimate of the transformable fraction of the pneumococcal cultures (see Text S2). For RMV7_rare_, the estimates are consistently close to ~1.5% of the population being transformable. For RMV7_wt_, the estimates are closer to 0.5%. This relatively small fold difference between the two variants reflects the disproportionate effect of GlcNAc on RMV7_wt_ relative to RMV7_rare_ (Fig. 3).


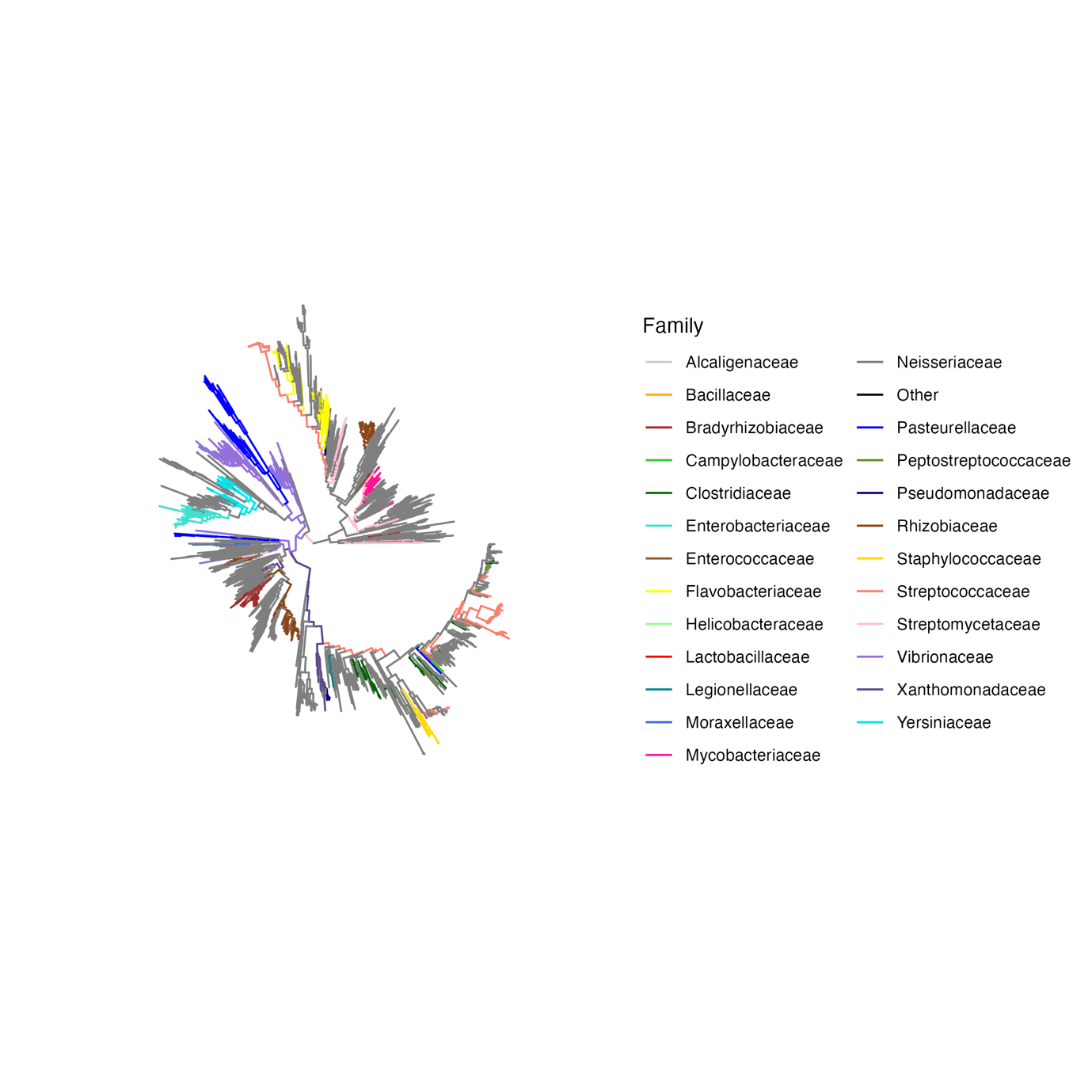


**Figure S48** Maximum likelihood phylogeny of 1,468 TfoX proteins. These sequences were identified using the EMBL SMART server and aligned with MAFFT. A phylogeny was generated with Fasttree2. Tips were assigned to families when they arose from a known taxon. These were used to partition and colour the phylogeny, according to the displayed legend, using ggtree. The clade shared by *H. influenzae* (Pasteurellaceae) and *V. cholerae* (Vibrionaceae) is distinct from that shared by *S. pneumoniae* (Streptococcaceae) and *Staphylococcus aureus* (Staphylococcaceae).


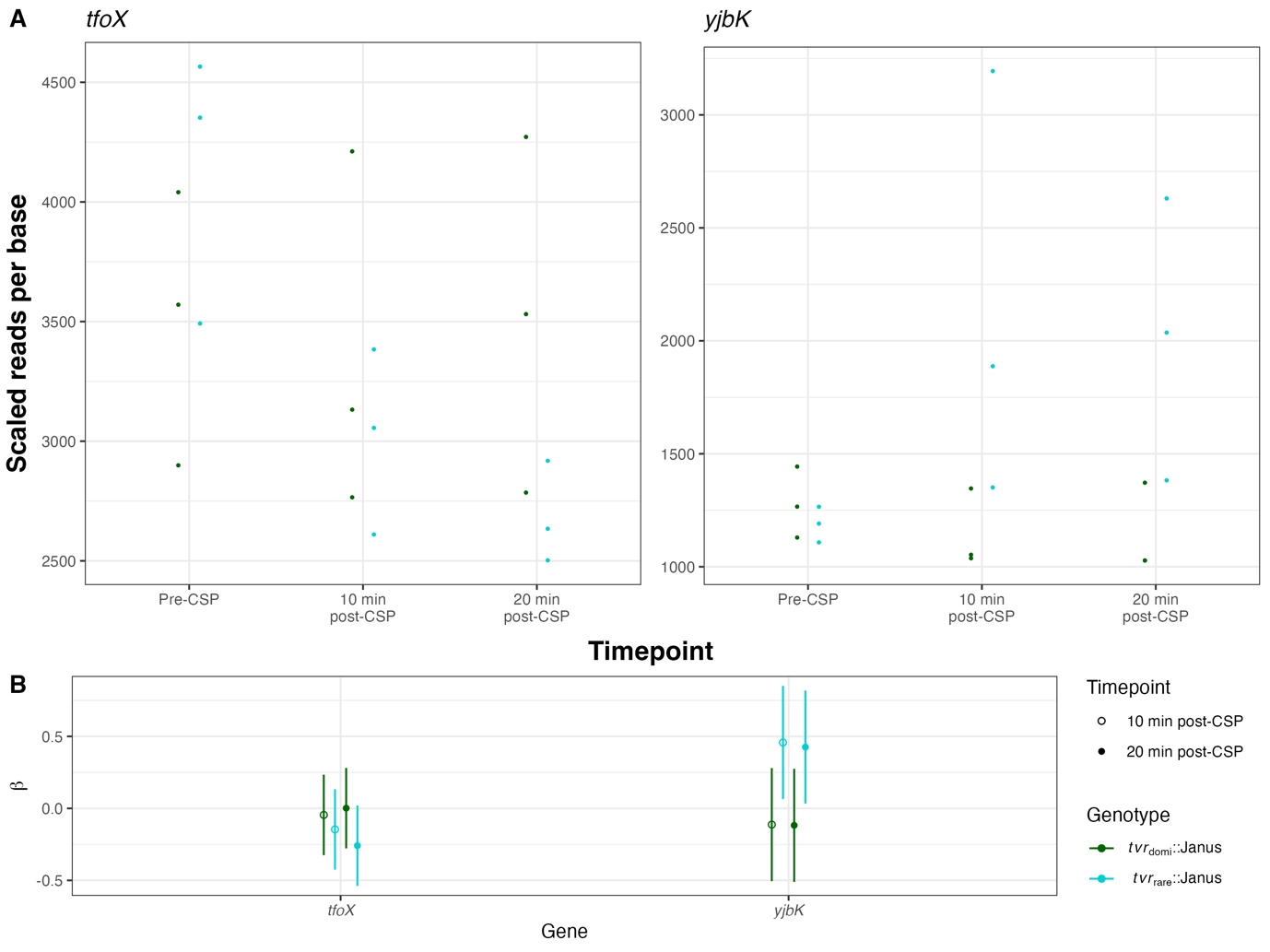


**Figure S49** Quantification of the expression of *yjbK* and *tfoX* using RNA-seq data. Data are displayed as in Fig. S11. Both genes are constitutively expressed, with the main difference between the variants being an apparent induction of *yjbK* expression after the addition of CSP.

**Supplementary Tables**

**Table S1** Genotypes constructed and analysed in this study.

| **Isolate name** | **List and description of genotypes** | **Reference or source (this study unless specified)** |
| --- | --- | --- |
| *S. pneumoniae* R6 | R6 *rpsL*Δivr*  R6 *rpsL*Δivr rpoB**  R6 *rpsL** Δ*ivr tfoX*::Janus  R6 *rpsL** Δ*ivr manLMN*::Janus  R6 *rpsL** Δ*ivr yjbK*::Janus  R6 *rpsL** Δ*ivr hrcA*::Janus  R6 *rpsL** Δ*ivr hrcA* restored | Apagyi *et al* 2018 |
| *S. pneumoniae* RMV7 | RMV7_wt_ (RMV7 *rpsL**)  RMV7_domi_ (RMV7 *rpsL* tvr*_TRDIII-iii_ *tvrR*::Janus)  RMV7_rare_ (RMV7 *rpsL* tvr*_TRDIII-i_ Δ*tvrR*)  RMV7_wt_ *rpsL** *tvr*::*cat*  RMV7 *tvr*_domi_::Janus (RMV7 *rpsL** *tvr*::*tvr*_TRDIII-iii_ *tvrR*::Janus)  RMV7 *tvr*_rare_::Janus (RMV7 *rpsL** *tvr*::*tvr*_TRDIII-i_ *tvrR*::Janus) | Kwun *et al* 2018  Kwun *et al* 2018  Kwun *et al* 2018 |
| RMV7_wt_ | RMV7_wt_ *phoB*::Janus  RMV7_wt_ *ciaRH*::Janus  RMV7_wt_ *htrA*::Janus  RMV7_wt_ *manLMN*::Janus  RMV7_wt_ *tfoX*::Janus  RMV7_wt_ *yjbK*::Janus  RMV7_wt_ *nagA*::Janus  RMV7_wt_ *rlrA*::Janus  RMV7_wt_ *rlrArrgABC*::Janus  RMV7_wt_ *rrgA*::Janus  RMV7_wt_ *rrgBC*::Janus  RMV7_wt_ PRCI*_dnaN_::*Janus  RMV7_wt_ PRCI*_dnaN+att_::*Janus  RMV7_wt_ PRCI*_reg-IONPJBJN00496_::*Janus  RMV7_wt_ PRCI*_rep_::*Janus  RMV7_wt_ PRCI*_int-rep_::*Janus  RMV7_wt_ PRCI*_reg_::*Janus  RMV7_wt_ PRCI*_dnaN+att_::*Janus *hrcA*::*cat*  RMV7_wt_ PRCI*_dnaN+att_::*Janus *clpP*::*cat*  RMV7_wt_ *clpP*::*cat*  RMV7_wt_ *clpP*::*cat hrcA::*Janus  RMV7_wt_ *hrcA*::*cat*  RMV7_wt_ *hrcA*::Janus |  |
| RMV7_rare_ | RMV7_rare_ *phoB*::Janus  RMV7_rare_ *phoB*::*phoB_wt_*  RMV7_rare_ *phoB* restored  RMV7_rare_ *piuA*::Janus  RMV7_rare_ *ciaRH*::Janus  RMV7_rare_ *htrA*::Janus  RMV7_rare_ *manLMN*::Janus  RMV7_rare_ *manL*::Janus  RMV7_rare_ *manL* restored  RMV7_rare_ *tfoX*::Janus  RMV7_rare_ *tfoX* restored  RMV7_rare_ *yjbK*::Janus  RMV7_rare_ *yjbK* restored  RMV7_rare_ *nagA*::Janus  RMV7_rare_ *manLMN*::Janus *tfoX*::*cat*  RMV7_rare_ *manLMN*::Janus *yjbK*::*cat*  RMV7_rare_ *yjbK*::Janus *tfoX*::*cat*  RMV7_rare_ *rlrA*::Janus  RMV7_rare_ *rlrA* restored  RMV7_rare_ *rlrArrgABC*::Janus  RMV7_rare_ *rrgA*::Janus  RMV7_rare_ *rrgBC*::Janus  RMV7_rare_ *hrcA*::Janus  RMV7_rare_ *hrcA* restored  RMV7_rare_ *clpP*::*cat*  RMV7_rare_ *clpP*::*cat hrcA::*Janus  RMV7_rare_ *hrcA*::Janus *manLMN*::*cat* |  |
| *S. pneumoniae* RMV5 | RMV7 *rpsL* tvr*_TRDIV-iii_ Δ*tvrR*  RMV7 *rpsL* tvr*_TRDI-i_ Δ*tvrR* | Kwun *et al* 2018  Kwun *et al* 2018 |
| *S. pneumoniae* RMV6 | RMV7 *rpsL* tvr*_TRDII-iii_ Δ*tvrR*  RMV7 *rpsL* tvr*_TRDIII-i_ Δ*tvrR* | Kwun *et al* 2018  Kwun *et al* 2018 |
| *S. pneumoniae* RMV8 | RMV7 *rpsL* tvr*_TRDI-i_ Δ*tvrR*  RMV7 *rpsL* tvr*_TRDIV-ii_ Δ*tvrR* | Kwun *et al* 2018  Kwun *et al* 2018 |
| *E. coli* | DH5α | Sigma |

**Table S2** Oligonucleotides used in this study

| **Name of oligonucleotide** | **Sequence** | **Function** |
| --- | --- | --- |
| hsdML | GCGGATGGTTTAAGTTTGGA | Checking arrangements of *tvr* locus |
| Trd III R v1 | TTCAATATAAAACTGCCATC | Checking arrangements of *tvr* locus |
| Trd IV R v2 | ATTTATCTCTGTTGAAGTTTTAGTCAG | Checking arrangements of *tvr* locus |
| Trd i R v1 | CTCCTCACTAAACAACTCATCTGA | Checking arrangements of *tvr* locus |
| Trd iii R v2 | TCCAAATGCTGTATCTACATTCCA | Checking arrangements of *tvr* locus |
| qRT rpoA For | TGGTCGTGGATATGTACCTGC | Transcriptional analysis |
| qRT rpoA Rev | CACGAGCAGGTTCCACTTGA | Transcriptional analysis |
| rpoB Full v2 For | GGCAGGACATGACGTTCAAT | Amplification of *rpoB** gene (conferring resistance to rifampicin) |
| rpoB Full v2 Rev | GCTTCCGCTTTTGTTGCTTT | Amplification of *rpoB** gene (conferring resistance to rifampicin) |
| qRT 507 For | AGGGAGAAAACTCTAATACCGT | Transcriptional analysis |
| qRT 507 For | GGCTTTTCTCAAGGTTTCAGGT | Transcriptional analysis |
| qRT hrcA For | TACCAAAACGCACGAACCTG | Transcriptional analysis |
| qRT hrcA Rev | AAAACCAGCAACACTTGGCA | Transcriptional analysis |
| qRT dnaK For | GGCTACGCTGAAGACTACCT | Transcriptional analysis |
| qRT dnaK Rev | CTGCTGCAGTTGGTTCGTTA | Transcriptional analysis |
| qRT manL For | GCGTGCCAATCAAGACTCTT | Transcriptional analysis |
| qRT manL Rev | ATTCAACACCCAAGTCACGC | Transcriptional analysis |
| qRT ciaR For | TGAACTGGGAGCGGATGATT | Transcriptional analysis |
| qRT ciaR Rev | ATCGAACTCTTTCCCCAGCA | Transcriptional analysis |
| qRT comYC For | TTGGTGGAGATGTTGGTGGT | Transcriptional analysis |
| qRT comYC Rev | TCGCCCATCTGCTTGTAACT | Transcriptional analysis |
| qRT comX For | TGGGAATTGTCGGATTGGGA | Transcriptional analysis |
| qRT comX Rev | CGCTTCTGACTTTCCTGCTT | Transcriptional analysis |
| qRT comEA For | AGGCTCTGGTTTACGTTCCT | Transcriptional analysis |
| qRT comEA Rev | TGTCCTGAGCTCGTTTTCCT | Transcriptional analysis |
| qRT comD For | AAGCAGATGGAAGTAGCAGTT | Transcriptional analysis |
| qRT comD Rev | ATCCGACTCCGCGATTTCTT | Transcriptional analysis |
| qRT htrA For | GCGGCCCACTGATCAATATT | Transcriptional analysis |
| qRT htrA rev | ACGCGTCACTTTTCCGTTTT | Transcriptional analysis |
| qRT groEL For | CGTGGACAAAGATAGCACGG | Transcriptional analysis |
| qRT groEL Rev | CCACCTGACAATTTGGCCAA | Transcriptional analysis |
| qRT clpP For | TTATGCTGACAGGTCCGGTT | Transcriptional analysis |
| qRT clpP Rev | CCCACTTGATGCGATGACAG | Transcriptional analysis |
| qRT clpE For | GCGCGACGATGAGATTATCC | Transcriptional analysis |
| qRT clpE Rev | TCGAATCCCCGTTCCTTGAA | Transcriptional analysis |
| phoB Up For | TAAGATTGGTGTCAAAGCAGGCCAGT | Removing *phoB* |
| phoB Up Rev ApaI | AAGGGCCCTTCTCTAATCATTTCCTCGTC | Removing *phoB* |
| phoB Down For BamHI | AAGGATCCGCTTTAGATTGTATCGTGAC | Removing *phoB* |
| phoB Down Rev | TATTCTCTAGGATAATTCTAAGACTGG | Removing *phoB* |
| phoB internal For | ATGATAGGCTGGTAGGAAATTTTC | Checking the sequence of *phoB* |
| htrA Up For | TCGAACCTGCGACCGTTCGCTTA | Removing *htrA* |
| htrA Up Rev ApaI | AAGGGCCCATGTTTCATATTTGCCTCCAT | Removing *htrA* |
| htrA Down For BamHI | AAGGATCCGAATCTTAATTGACATCTATG | Removing *htrA* |
| htrA Down Rev | AATGTTACCAAACTTTATCCACAGGTT | Removing *htrA* |
| hrcA Up For | GGCGTAAATTTTGCTATAATATTTTCG | Removing *hrcA* |
| hrcA Up Rev ApaI | AAGGGCCCGCTAGAGTTAATAGACTCTTGC | Removing *hrcA* |
| hrcA Down For BamHI | AAGGATCCAATCAAGTCAATGTGGTCAAC | Removing *hrcA* |
| hrcA Down Rev | TGGTTCGCTTCGCTCACTATTACAAA | Removing *hrcA* |
| yjbK Up For | CACGATAATCTGTATAGAAGCCCAAATGT | Removing *yjbK* |
| yjbK Up Rev ApaI | AAGGGCCCCAATAGTGTTTTCAATTCAAT | Removing *yjbK* |
| yjbK Down For BamHI | AAGGATCCAGTATGAAAAATAGCTGAAAT | Removing *yjbK* |
| yjbK Down Rev | ATATTAGTATTGTCAAGAAGCTCAGCAGC | Removing *yjbK* |
| ciaRH Up For | CGTCATAACTATGACCGTCTTGGTTTTG | Removing *ciaRH* |
| ciaRH Up Rev ApaI | AAGGGCCCCAGACCTAGGTCATCCTCAA | Removing *ciaRH* |
| ciaRH Down For BamHI | AAGGATCCAATCTTTGAAGTGAAGATTGC | Removing *ciaRH* |
| ciaRH Down Rev | CCAGAAGTTAATCTGACGACTGCTAGT | Removing *ciaRH* |
| tvr Up For | ACAACCTTTGACTACGCCTATACACC | Removing *tvr* locus |
| tvr Up Rev ApaI | AAGGGCCCATCTTCCCTTTTCTTTAGTT | Removing *tvr* locus |
| tvr Down For BamHI | AAGATATCATTCTAAAGTGATTGCCATGC | Removing *tvr* locus |
| tvr Down Rev | AAGCCCTTCTTGCTAAGAACGACATTC | Removing *tvr* locus |
| tfoX Up For | GGCGCAAAATACGACCATCATG | Removing *tfoX* |
| tfoX Up Rev ApaI | AAGGGCCCGTTCTTACTTGATGCCAT | Removing *tfoX* |
| tfoX Down For BamHI | AAGGATCCGAAATGATTTGAGTGGAAG | Removing *tfoX* |
| tfoX Down Rev | TGCAAAAGCACCGGAATTGGTGA | Removing *tfoX* |
| manLMN Up For | GCCAAATCCAAGGACGTATGGTACTCGA | Removing *manLMN* |
| manLMN Up Rev ApaI | AAGGGCCCCATCACGCGACTAGCTTGGTT | Removing *manLMN* |
| manLMN Down For BamHI | AAGGATCCAACTTGATTCGTATGTCATTC | Removing *manLMN* |
| manLMN Down Rev | AGGAATCAGATTTTAGAACCTGGTTCC | Removing *manLMN* |
| manL Down For BamHI | AAGGATCCAACAAAGCCAATGTCAAATAA | Removing *manL* |
| manL Down Rev | TCCAAGACCCTTGTAAGAAGGTTG | Removing *manL* |
| piuA Up For | TAAGGATAAGAGGTCCCACTTAAAAC | Removing *piuA* |
| piuA Up Rev ApaI | AAGGGCCCAGAATTAGAAGAACAAGCGCT | Removing *piuA* |
| piuA Down For BamHI | AAGGATCCGTAATTCCAGATAATACACCG | Removing *piuA* |
| piuA Down Rev | CTTGCAATTATTGCAAGTGATCTTATC | Removing *piuA* |
| nagA Up For | ACAGACTTAACTGTCAATGGCTTGAAG | Removing *nagA* |
| nagA Up Rev ApaI | AAGGGCCCCACAAGTTCCAAGTAACCA | Removing *nagA* |
| nagA Down For BamHI | AAGGATCCGCTAAATCCGTTCACATCGA | Removing *nagA* |
| nagA Down Rev | CGTCTAGGTCTACCATAATCAATTTAGC | Removing *nagA* |
| rlrA Up For | CATTCTTCTCAACTTCAACAGTCCATT | Removing *rlrA* |
| rlrA Up Rev ApaI | AAGGGCCCCAGTTCATCTAGTTCAATAG | Removing *rlrA* |
| rlrA Down For BamHI | AAGGATCCTTAGATTTGCATGTTAGCCA | Removing *rlrA* |
| rlrA Down Rev | CTGAGCAATGAAAGCCAATTTCCC | Removing *rlrA* |
| rrgA Up For | GAAATATACTCTTTTTGTGTAAATTC | Removing *rrgA* |
| rrgA Up Rev ApaI | AAGGGCCCTTCAGGCGTTTCTGCTAAA | Removing *rrgA* |
| rrgA Down For BamHI | AAGGATCCACAGGTGAAGATGGTAAG | Removing *rrgA* |
| rrgA Down Rev | TTTTCATCAATAATTTCATTATTAG | Removing *rrgA* |
| rrgBC Up For | CCTAAGGAAGTAGAAAAGAACACAGTG | Removing *rrgBC* |
| rrgBC Up Rev ApaI | AAGGGCCCGGCAGCAAGCATTGTTAAAAA | Removing *rrgBC* |
| rrgBC Down For BamHI | AAGGATCCTTGATGCTTGTTGCCATTTTG | Removing *rrgBC* |
| rrgBC Down Rev | GGTCAAATCCGTAAACATCTTAGCCGT | Removing *rrgBC* |
| clpP Up For | AATTGAAGTTATTAATCACCCACTGA | Removing *clpP* |
| clpP Up Rev ApaI | AAGGGCCCAGAACGTTCTCCACGGCTTGT | Removing *clpP* |
| clpP Down For BamHI | AAGGATCCAGCGCCCAGGAAACACTTGAA | Removing *clpP* |
| clpP Down Rev | AATGGAACACCTGCTTTTGTAGCGTTC | Removing *clpP* |
| PRCI Up For v2 | TCGTAATTCCTAGCCGTTCTCTACGCG | Removing PRCI with *att* sites |
| PRCI Up Rev ApaI v2 | AAGGGCCCTCAGTCAATAATTTTATTCA | Removing PRCI with *att* sites |
| PRCI Down For BamHI v2 | AAGGATCCGTGTTATAATATTAGGGATTG | Removing PRCI with *att* sites |
| PRCI Down Rev v2 | TTCTTCCTCAGCACGCGCAGAAATAAC | Removing PRCI with *att* sites |
| PRCI Up For | CCAACGATACCTGCTGTCA | Removing PRCI |
| PRCI Up Rev ApaI | AAGGGCCCAGCAGGGTAAATACGGCAGA | Removing PRCI |
| PRCI Down For BamHI | AAGGATCCCTTTAACCCTGCCCGTGATG | Removing PRCI |
| PRCI Down Rev | AAAAGGCTAATCGTTGGGAAATT | Removing PRCI |
| PRCI Regulator Up For | GTATACAACCGTCAACGATTGGGTAA | Removing PRCI’s regulator |
| PRCI Regulator Up Rev ApaI | AAGGGCCCGTGTATTCTATATGTACGATG | Removing PRCI’s regulator |
| PRCI Regulator For BamHI | AAGGATCCCGAATAACATCTAACGATTTT | Removing PRCI’s regulator |
| PRCI Regulator Down Rev | CGCCCTTGAGTCTTTTGGGATGATGAT | Removing PRCI’s regulator |
| dinD Up For | CCCTATTCCGATGAAAATACTGTTAAC | Removing IONPJBJN_00496 |
| dinD Up Rev ApaI | AAGGGCCCAATAAAGTATCCTTTCTAAAA | Removing IONPJBJN_00496 |
| tvrR Inf Up For | AATCACCATTACGATTCCAAGTGAATTT | Removing N-terminal 267bp of *tvrR* |
| tvrR Inf Up Rev ApaI | GGGGGCCCAATTTCTCACTTTCTTATTCA | Removing N-terminal 267bp of *tvrR* |
| tvrR Inf Up R BamHI | TTGGATCCAATTTCTCACTTTCTTATTCA | Removing Janus from *tvrR*::Janus mutant |
| tvrR Inf Down For BamHI | TTGGATCCTTAAGGAGTTATTTAGCAAATT | Removing N-terminal 267bp of *tvrR* |
| tvrR Inf Down Rev | CTTTTAAGAGATGAATTTTTGGTGTGA | Removing N-terminal 267bp of *tvrR* |
| tvrR Up For | AATCACCATTACGATTCCAAGTGAATTT | Removing C-terminal 681bp of *tvrR* |
| tvrR Up Rev ApaI | TTGGGCCCACTACAATCCTTTTCAGACTG | Removing C-terminal 681bp of *tvrR* |
| tvrR Down For BamHI | TTGGATCCAAGCAAATCCCGATATTCCGA | Removing C-terminal 681bp of *tvrR* |
| Janus For ApaI | TTGGGCCCCCGTTTGATTTTTAATGGATAATGTG | Amplifying Janus |
| Janus Rev BamHI | ATGGATCCCCTTTCCTTATGCTTTTGGACG | Amplifying Janus |
| Cat For ApaI | AAGGGCCCAGTGGGATATTTTTAAAATAT | Amplifying *cat* |
| Cat Rev BamHI | AAGGATCCTTATAAAAGCCAGTCATTAGG | Amplifying *cat* |

**Table S3** Single nucleotide polymorphisms distinguishing RMV7_domi_ and RMV7_rare_. All positions are relative to the annotation of RMV7_domi_ (accession code OV904788). The alleles in RMV7_wt_ were inferred from the assembly of the original isolate (accession code GCA_001091925).

| **Position RMV7_domi_** | **Allele in RMV7_domi_** | **Allele in RMV7_rare_** | **Allele in RMV7_wt_** | **Type** | **Affected gene** | **Description** |
| --- | --- | --- | --- | --- | --- | --- |
| 420138 | G | A | G | Non-synonymous | IONPJBJN_00426 (*ktrA*) | Non-synonymous mutation in potassium transporter |
| 404703 | A | . | A | Intergenic | - | Mutation upstream of gene encoding hypothetical protein |
| 371741 | . | C | . | Intergenic | - | Mutation in homopolymeric tract upstream of lytic amidase |
| 211954 | A | G | G | Non-synonymous | IONPJBJN_00199 (*xerC_2)* | Non-synonymous mutation in mobile element integrase |
| 120592 | A | G | G | Premature stop | IONPJBJN_00114 | Premature stop codon affecting membrane protein in RMV7_domi_ |
| 1833163 | G | A | G | Premature stop | IONPJBJN_01863 (*pstS1*) | Premature stop codon in RMV7_rare_ |
| 1467555 | A | C | C | Synonymous | IONPJBJN_01511 (*uvrC*) | Synonymous change in gene encoding DNA repair protein |
| 1376435 | T | C | T | Variation in *tvr* locus | IONPJBJN_01427 | Variation in rearranged *hsdS* gene |
| 1376377 | G | A | G | Variation in *tvr* locus | IONPJBJN_01427 | Variation in rearranged *hsdS* gene |
| 1376374 | A | C | A | Variation in *tvr* locus | IONPJBJN_01427 | Variation in rearranged *hsdS* gene |
| 1376335 | A | G | A | Variation in *tvr* locus | IONPJBJN_01427 | Variation in rearranged *hsdS* gene |
| 1376274 | C | G | C | Variation in *tvr* locus | IONPJBJN_01427 | Variation in rearranged *hsdS* gene |
| 1376039 | C | T | C | Variation in *tvr* locus | IONPJBJN_01426 (*xerC_3*) | Variation in *tvrR* gene within rearranged *tvr* locus |
| 1375985 | A | G | A | Variation in *tvr* locus | IONPJBJN_01426 (*xerC_3*) | Variation in *tvrR* gene within rearranged *tvr* locus |
| 1375965 | C | T | C | Variation in *tvr* locus | IONPJBJN_01426 (*xerC_3*) | Variation in *tvrR* gene within rearranged *tvr* locus |
| 1375943 | T | A | T | Variation in *tvr* locus | IONPJBJN_01426 (*xerC_3*) | Variation in *tvrR* gene within rearranged *tvr* locus |
| 1375892 | T | C | T | Variation in *tvr* locus | IONPJBJN_01426 (*xerC_3*) | Variation in *tvrR* gene within rearranged *tvr* locus |
| 1375728 | A | G | G | Variation in *tvr* locus | IONPJBJN_01426 (*xerC_3*) | Variation in *tvrR* gene within rearranged *tvr* locus |
| 1375614 | T | C | T | Variation in *tvr* locus | IONPJBJN_01426 (*xerC_3*) | Variation in *tvrR* gene within rearranged *tvr* locus |
| 1187336 | C | A | A | Non-synonymous | IONPJBJN_01237 (*clpX*) | Non-synonymous mutation in gene encoding a protease |
| 931762 | C | A | C | Non-synonymous | IONPJBJN_00977 (*feuB*) | Non-synonymous mutation in *piaA* iron transporter gene |
| 751099 | G | T | G | Non-synonymous | IONPJBJN_00765 | Non-synonymous mutation in gene encoding an ion transporter protein |
| 697306 | T | C | T | Non-synonymous | IONPJBJN_00700 (*gltX*) | Synonymous change in gene encoding glutamate--tRNA ligase |
| 561121 | T | C | T | Non-synonymous | IONPJBJN_00578 (*phoB*) | Non-synonymous mutation in gene encoding a regulatory protein |

**Table S4** Accession codes for RNA-seq datasets.

| **Accession code** | **Genotype** | **Timepoint** |
| --- | --- | --- |
| ERS3382236 | RMV7 *tvr*_rare_::Janus | Pre-CSP |
| ERS3382237 | RMV7 *tvr*_rare_::Janus | 10 min post-CSP |
| ERS3382238 | RMV7 *tvr*_rare_::Janus | 20 min post-CSP |
| ERS3382239 | RMV7 *tvr*_domi_::Janus | Pre-CSP |
| ERS3382240 | RMV7 *tvr*_domi_::Janus | 10 min post-CSP |
| ERS3382241 | RMV7 *tvr*_domi_::Janus | 20 min post-CSP |
| ERS3382242 | RMV7 *tvr*_rare_::Janus | Pre-CSP |
| ERS3382243 | RMV7 *tvr*_rare_::Janus | 10 min post-CSP |
| ERS3382244 | RMV7 *tvr*_rare_::Janus | 20 min post-CSP |
| ERS3382227 | RMV7 *tvr*_domi_::Janus | Pre-CSP |
| ERS3382228 | RMV7 *tvr*_domi_::Janus | 10 min post-CSP |
| ERS3382229 | RMV7 *tvr*_domi_::Janus | 20 min post-CSP |
| ERS3382230 | RMV7 *tvr*_rare_::Janus | Pre-CSP |
| ERS3382231 | RMV7 *tvr*_rare_::Janus | 10 min post-CSP |
| ERS3382232 | RMV7 *tvr*_rare_::Janus | 20 min post-CSP |
| ERS3382233 | RMV7 *tvr*_domi_::Janus | Pre-CSP |
| ERS3382234 | RMV7 *tvr*_domi_::Janus | 10 min post-CSP |
| ERS3382235 | RMV7 *tvr*_domi_::Janus | 20 min post-CSP |

**Table S5** Differential gene expression analysis (see spreadsheet)

**Table S6** Logistic model parameters estimated from growth curves.

| **Genotype** | **Condition** | **Carrying capacity estimate, *K*** | **Carrying capacity standard error** | **Growth rate estimate, *r* (h^-1^)** | **Growth rate standard error (h^-1^)** | **Figure** |
| --- | --- | --- | --- | --- | --- | --- |
| RMV7_rare_ | Aminoglucose | 0.734 | 0.001 | 1.206 | 0.009 | Fig. S26 |
| RMV7_rare_ | Galactose | 0.719 | 0.002 | 1.292 | 0.028 | Fig. S26 |
| RMV7_rare_ | Glucose | 0.718 | 0.006 | 1.624 | 0.115 | Fig. S26 |
| RMV7_rare_ | Mannose | 0.616 | 0.006 | 1.352 | 0.104 | Fig. S26 |
| RMV7_rare_ | N-acetylglucosamine | 0.756 | 0.004 | 1.472 | 0.064 | Fig. S26 |
| RMV7_rare_ | None | 0.687 | 0.003 | 1.552 | 0.055 | Fig. S26 |
| RMV7_rare_ | Sialic acid | 0.718 | 0.004 | 1.114 | 0.041 | Fig. S26 |
| RMV7_rare_ *manLMN*::Janus | Aminoglucose | 0.801 | 0.005 | 1.252 | 0.062 | Fig. S26 |
| RMV7_rare_ *manLMN*::Janus | Galactose | 0.773 | 0.006 | 1.265 | 0.077 | Fig. S26 |
| RMV7_rare_ *manLMN*::Janus | Glucose | 0.750 | 0.007 | 1.350 | 0.106 | Fig. S26 |
| RMV7_rare_ *manLMN*::Janus | Mannose | 0.738 | 0.005 | 1.261 | 0.073 | Fig. S26 |
| RMV7_rare_ *manLMN*::Janus | N-acetylglucosamine | 0.784 | 0.004 | 1.001 | 0.039 | Fig. S26 |
| RMV7_rare_ *manLMN*::Janus | None | 0.833 | 0.006 | 1.155 | 0.058 | Fig. S26 |
| RMV7_rare_ *manLMN*::Janus | Sialic acid | 0.647 | 0.005 | 1.172 | 0.073 | Fig. S26 |
| RMV7_wt_ | Aminoglucose | 0.327 | 0.003 | 0.871 | 0.043 | Fig. S26 |
| RMV7_wt_ | Galactose | 0.379 | 0.002 | 1.064 | 0.040 | Fig. S26 |
| RMV7_wt_ | Glucose | 0.348 | 0.006 | 1.509 | 0.231 | Fig. S26 |
| RMV7_wt_ | Mannose | 0.435 | 0.003 | 0.946 | 0.043 | Fig. S26 |
| RMV7_wt_ | N-acetylglucosamine | 0.497 | 0.002 | 1.290 | 0.044 | Fig. S26 |
| RMV7_wt_ | None | 0.370 | 0.005 | 1.083 | 0.102 | Fig. S26 |
| RMV7_wt_ | Sialic acid | 0.324 | 0.001 | 1.023 | 0.032 | Fig. S26 |
| RMV7_wt_ *manLMN*::Janus | Aminoglucose | 0.518 | 0.004 | 0.725 | 0.029 | Fig. S26 |
| RMV7_wt_ *manLMN*::Janus | Galactose | 0.542 | 0.007 | 1.109 | 0.099 | Fig. S26 |
| RMV7_wt_ *manLMN*::Janus | Glucose | 0.515 | 0.009 | 1.081 | 0.126 | Fig. S26 |
| RMV7_wt_ *manLMN*::Janus | Mannose | 0.490 | 0.007 | 0.964 | 0.087 | Fig. S26 |
| RMV7_wt_ *manLMN*::Janus | N-acetylglucosamine | 0.535 | 0.008 | 0.855 | 0.066 | Fig. S26 |
| RMV7_wt_ *manLMN*::Janus | None | 0.571 | 0.010 | 1.050 | 0.114 | Fig. S26 |
| RMV7_wt_ *manLMN*::Janus | Sialic acid | 0.530 | 0.007 | 0.880 | 0.067 | Fig. S26 |
| RMV7_rare_ | Aminoglucose | 0.734 | 0.001 | 1.206 | 0.009 | Fig. S26 |
| RMV7_rare_ | Galactose | 0.719 | 0.002 | 1.292 | 0.028 | Fig. S26 |
| RMV7_rare_ | Glucose | 0.718 | 0.006 | 1.624 | 0.115 | Fig. S26 |
| RMV7_rare_ | Mannose | 0.616 | 0.006 | 1.352 | 0.104 | Fig. S26 |
| RMV7_rare_ | N-acetylglucosamine | 0.756 | 0.004 | 1.472 | 0.064 | Fig. S26 |
| RMV7_rare_ |  | 0.740 | 0.003 | 1.511 | 0.053 | Fig. S36 |
| RMV7_rare_ *nagA*::Janus |  | 0.491 | 0.013 | 1.764 | 0.446 | Fig. S36 |
| RMV7_rare_ *tfoX*::Janus |  | 0.713 | 0.004 | 2.005 | 0.135 | Fig. S36 |
| RMV7_rare_ *yjbK*::Janus |  | 0.736 | 0.004 | 2.022 | 0.119 | Fig. S36 |
| RMV7_rare_ *nagA*::Janus | Aminoglucose | 0.495 | 0.005 | 1.080 | 0.080 | Fig. S37 |
| RMV7_rare_ *nagA*::Janus | Galactose | 0.490 | 0.005 | 1.231 | 0.089 | Fig. S37 |
| RMV7_rare_ *nagA*::Janus | Glucose | 0.522 | 0.008 | 1.234 | 0.145 | Fig. S37 |
| RMV7_rare_ *nagA*::Janus | Mannose | 0.351 | 0.023 | 1.240 | 0.031 | Fig. S37 |
| RMV7_rare_ *nagA*::Janus | N-acetylglucosamine | 367.534 | 3.46x10^9^ | 0.040 | 0.135 | Fig. S37 |
| RMV7_rare_ *nagA*::Janus | None | 0.401 | 0.010 | 1.611 | 0.396 | Fig. S37 |
| RMV7_rare_ *nagA*::Janus | Sialic acid | 0.329 | 0.011 | 1.979 | 0.723 | Fig. S37 |
| RMV7_rare_ *tfoX*::Janus | Aminoglucose | 0.753 | 0.003 | 1.525 | 0.052 | Fig. S37 |
| RMV7_rare_ *tfoX*::Janus | Galactose | 0.727 | 0.002 | 1.239 | 0.028 | Fig. S37 |
| RMV7_rare_ *tfoX*::Janus | Glucose | 0.738 | 0.031 | 0.418 | 0.043 | Fig. S37 |
| RMV7_rare_ *tfoX*::Janus | Mannose | 0.658 | 0.006 | 1.178 | 0.035 | Fig. S37 |
| RMV7_rare_ *tfoX*::Janus | N-acetylglucosamine | 0.737 | 0.002 | 1.586 | 0.046 | Fig. S37 |
| RMV7_rare_ *tfoX*::Janus | None | 0.675 | 0.003 | 1.731 | 0.074 | Fig. S37 |
| RMV7_rare_ *tfoX*::Janus | Sialic acid | 0.654 | 0.003 | 1.291 | 0.047 | Fig. S37 |
| RMV7_rare_ *yjbK*::Janus | Aminoglucose | 0.803 | 0.002 | 1.469 | 0.035 | Fig. S37 |
| RMV7_rare_ *yjbK*::Janus | Galactose | 0.725 | 0.002 | 1.320 | 0.021 | Fig. S37 |
| RMV7_rare_ *yjbK*::Janus | Glucose | 0.673 | 0.014 | 0.544 | 0.045 | Fig. S37 |
| RMV7_rare_ *yjbK*::Janus | Mannose | 0.653 | 0.005 | 1.424 | 0.067 | Fig. S37 |
| RMV7_rare_ *yjbK*::Janus | N-acetylglucosamine | 0.738 | 0.002 | 1.763 | 0.053 | Fig. S37 |
| RMV7_rare_ *yjbK*::Janus | None | 0.659 | 0.002 | 1.675 | 0.044 | Fig. S37 |
| RMV7_rare_ *yjbK*::Janus | Sialic acid | 0.662 | 0.003 | 1.349 | 0.045 | Fig. S37 |
| RMV7_rare_ |  | 0.687 | 0.003 | 1.549 | 0.060 | Fig. S40 |
| RMV7_rare_ *rlrA*::Janus |  | 0.649 | 0.004 | 1.775 | 0.098 | Fig. S40 |
| RMV7_rare_ rrg*A*::Janus |  | 0.691 | 0.004 | 1.698 | 0.080 | Fig. S40 |
| RMV7_rare_ *rrgBC*::Janus |  | 0.659 | 0.006 | 1.829 | 0.155 | Fig. S40 |
| RMV7_wt_ |  | 0.488 | 0.009 | 1.174 | 0.162 | Fig. S41 |
| RMV7_wt_ PRCI*_dnaN_*::Janus |  | 0.559 | 0.011 | 1.123 | 0.172 | Fig. S41 |
| RMV7_rare_ | 35 °C | 0.676 | 0.004 | 1.688 | 0.097 | Fig. S43 |
| RMV7_rare_ | 40 °C | 0.266 | 0.023 | 9.594 | 67.500 | Fig. S43 |
| RMV7_rare_ *clpP*::*cat* | 35 °C | 0.594 | 0.008 | 1.744 | 0.228 | Fig. S43 |
| RMV7_rare_ *clpP*::*cat* | 40 °C | 0.124 | 0.079 | 0.086 | 0.217 | Fig. S43 |
| RMV7_rare_ *clpP*::*cat* *hrcA*::Janus | 35 °C | 0.485 | 0.013 | 2.482 | 0.764 | Fig. S43 |
| RMV7_rare_ *clpP*::*cat* *hrcA*::Janus | 40 °C | 0.017 | 0.115 | 0.013 | 0.100 | Fig. S43 |
| RMV7_rare_ *hrcA*::Janus | 35 °C | 0.501 | 0.011 | 2.719 | 0.692 | Fig. S43 |
| RMV7_rare_ *hrcA*::Janus | 40 °C | 0.031 | 0.143 | 0.021 | 0.126 | Fig. S43 |
| RMV7_wt_ | 35 °C | 0.429 | 0.008 | 1.315 | 0.203 | Fig. S43 |
| RMV7_wt_ | 40 °C | 0.009 | 0.051 | 0.015 | 0.095 | Fig. S43 |
| RMV7_wt_ *clpP*::*cat* | 35 °C | 0.353 | 0.004 | 0.929 | 0.070 | Fig. S43 |
| RMV7_wt_ *clpP*::*cat* | 40 °C | 0.004 | 0.051 | 0.009 | 0.123 | Fig. S43 |
| RMV7_wt_ *clpP*::*cat* *hrcA*::Janus | 35 °C | 0.212 | 0.016 | 2.160 | 1.801 | Fig. S43 |
| RMV7_wt_ *clpP*::*cat* *hrcA*::Janus | 40 °C | 0.017 | 0.001 | 0.216 | 0.033 | Fig. S43 |
| RMV7_wt_ *hrcA*::Janus | 35 °C | 0.450 | 0.003 | 1.610 | 0.107 | Fig. S43 |
| RMV7_wt_ *hrcA*::Janus | 40 °C | 0.042 | 0.001 | 0.273 | 0.012 | Fig. S43 |

**Table S7** Correspondence between figures and files available from <https://figshare.com/projects/Diverse_regulatory_pathways_modulate_bet_hedging_of_competence_induction_in_epigenetically-differentiated_phase_variants_of_Streptococcus_pneumoniae/171060>.

| **Figure** | **Dataset** | **File** |
| --- | --- | --- |
| 1A | [Experimental data from the analysis of RMV7 phase variants](https://figshare.com/account/projects/171060/articles/23634165) | Comparison_of_RMV_phase_variants.csv |
| 1C | [Experimental data from the analysis of RMV7 phase variants](https://figshare.com/account/projects/171060/articles/23634165) | RMV7_phase_variant_frequencies.csv |
| 1D | [Experimental data from the analysis of RMV7 phase variants](https://figshare.com/account/projects/171060/articles/23634165) | RMV7_domi_rare_wt_transformation_frequencies.csv |
| 1E | [Experimental data from the analysis of RMV7 phase variants](https://figshare.com/account/projects/171060/articles/23634165) | RMV7_domi_rare_wt_biofilm_thicknesses.csv |
| 1F | [Experimental data from the analysis of RMV7 phase variants](https://figshare.com/account/projects/171060/articles/23634165) | RMV7_tvr_domi_rare_passage_transformation_frequencies.csv |
| 2 | [RNA-seq data from a comparison of RMV7 epigenetic phase variants](https://figshare.com/articles/dataset/RNA-seq_data_from_a_comparison_of_RMV7_epigenetic_phase_variants/23589270) | RMV7_RNA_seq_statistics.csv |
| 3A | [Experimental data from the analysis of RMV7 phase variants](https://figshare.com/account/projects/171060/articles/23634165) | RMV7_rare_carbohydrates_transformation_efficiency.csv |
| 3B | [Experimental data from the analysis of RMV7 phase variants](https://figshare.com/account/projects/171060/articles/23634165) | RMV7_wt_carbohydrates_transformation_efficiency.csv |
| 3C | [Experimental data from the analysis of RMV7 phase variants](https://figshare.com/account/projects/171060/articles/23634165) | RMV7_rare_carbohydrates_biofilm.csv |
| 3D | [Experimental data from the analysis of RMV7 phase variants](https://figshare.com/account/projects/171060/articles/23634165) | RMV7_rare_GlcNAc_double_mutants_transformation_efficiencies.csv |
| 3E | [Experimental data from the analysis of RMV7 phase variants](https://figshare.com/account/projects/171060/articles/23634165) | Comparison_of_RMV_phase_variants_in_GlcNAc.csv |
| 4A | [Experimental data from the analysis of RMV7 phase variants](https://figshare.com/account/projects/171060/articles/23634165) | RMV7_wt_PRCI_mutants.csv |
| 4B | [Experimental data from the analysis of RMV7 phase variants](https://figshare.com/account/projects/171060/articles/23634165) | RMV7_wt_PRCI_chaperone_mutants.csv |
| 4C | [Experimental data from the analysis of RMV7 phase variants](https://figshare.com/account/projects/171060/articles/23634165) | RMV7_wt_PRCI_mutant_gene_expression.csv |
| 4D | [Experimental data from the analysis of RMV7 phase variants](https://figshare.com/account/projects/171060/articles/23634165) | RMV7_wt_PRCI_mutant_gene_expression.csv |
| 5A | [Experimental data from the analysis of RMV7 phase variants](https://figshare.com/account/projects/171060/articles/23634165) | RMV7_heat_shock_transformation.csv |
| 5B | [Experimental data from the analysis of RMV7 phase variants](https://figshare.com/account/projects/171060/articles/23634165) | RMV7_rare_CaCl2_titration.csv |
| 5C | [Experimental data from the analysis of RMV7 phase variants](https://figshare.com/account/projects/171060/articles/23634165) | RMV7_rare_CaCl2_effect_on_hrcA.csv |
| 5D | [Experimental data from the analysis of RMV7 phase variants](https://figshare.com/account/projects/171060/articles/23634165) | RMV7_rare_comEAX_expression.csv |
| 5E | [Experimental data from the analysis of RMV7 phase variants](https://figshare.com/account/projects/171060/articles/23634165) | RMV7_rare_manLMN_hrcA_double_mutants.csv |
| S1 | [Agarose gel electrophoresis separation of PCR amplicons from RMV7 tvr loci](https://figshare.com/account/projects/171060/articles/23633925) | RMV7_wt_tvr_variation.jpg |
| S1 | [Agarose gel electrophoresis separation of PCR amplicons from RMV7 tvr loci](https://figshare.com/account/projects/171060/articles/23633925) | RMV7_domi_rare_tvr_domi_tvr_rare_tvr_arrangements.jpg |
| S2A | [Experimental data from the analysis of RMV7 phase variants](https://figshare.com/account/projects/171060/articles/23634165) | RMV7_wt_spontaneous_resistance_frequency.csv |
| S2B | [Experimental data from the analysis of RMV7 phase variants](https://figshare.com/account/projects/171060/articles/23634165) | RMV7_overnight_transformation_efficiencies.csv |
| S3 | [Morphology of RMV7 variant colonies](https://figshare.com/account/projects/171060/articles/23633865) | RMV7_domi_x10_magnification.jpg |
| S3 | [Morphology of RMV7 variant colonies](https://figshare.com/account/projects/171060/articles/23633865) | RMV7_domi_x4_magnification.jpg |
| S3 | [Morphology of RMV7 variant colonies](https://figshare.com/account/projects/171060/articles/23633865) | RMV7_rare_x10_magnification.jpg |
| S3 | [Morphology of RMV7 variant colonies](https://figshare.com/account/projects/171060/articles/23633865) | RMV7_rare_x4_magnification.jpg |
| S3 | [Morphology of RMV7 variant colonies](https://figshare.com/account/projects/171060/articles/23633865) | RMV7_wt_x10_magnification.jpg |
| S3 | [Morphology of RMV7 variant colonies](https://figshare.com/account/projects/171060/articles/23633865) | RMV7_wt_x4_magnification.jpg |
| S4A | [Experimental data from the analysis of RMV7 phase variants](https://figshare.com/account/projects/171060/articles/23634165) | RMV7_phoB_transformation_efficiency.csv |
| S4B | [Experimental data from the analysis of RMV7 phase variants](https://figshare.com/account/projects/171060/articles/23634165) | RMV7_pstS_transformation_efficiency.csv |
| S5B | [Agarose gel electrophoresis separation of PCR amplicons from RMV7 tvr loci](https://figshare.com/account/projects/171060/articles/23633925) | RMV7_domi_rare_tvr_domi_tvr_rare_tvr_arrangements.jpg |
| S5C | [Experimental data from the analysis of RMV7 phase variants](https://figshare.com/account/projects/171060/articles/23634165) | RMV7_tvr_mutant_transformation_efficiencies.csv |
| S5D | [Experimental data from the analysis of RMV7 phase variants](https://figshare.com/account/projects/171060/articles/23634165) | RMV7_tvr_mutant_biofilm_thicknesses.csv |
| S6 | [RNA-seq data from a comparison of RMV7 epigenetic phase variants](https://figshare.com/articles/dataset/RNA-seq_data_from_a_comparison_of_RMV7_epigenetic_phase_variants/23589270) | RMV7_RNA_seq_fragment_lengths.csv |
| S7 | [RNA-seq data from a comparison of RMV7 epigenetic phase variants](https://figshare.com/articles/dataset/RNA-seq_data_from_a_comparison_of_RMV7_epigenetic_phase_variants/23589270) | RMV7_RNA_seq_expression.csv |
| S8 | [RNA-seq data from a comparison of RMV7 epigenetic phase variants](https://figshare.com/articles/dataset/RNA-seq_data_from_a_comparison_of_RMV7_epigenetic_phase_variants/23589270) | RMV7_RNA_seq_statistics.csv |
| S9 | [RNA-seq data from a comparison of RMV7 epigenetic phase variants](https://figshare.com/articles/dataset/RNA-seq_data_from_a_comparison_of_RMV7_epigenetic_phase_variants/23589270) | RMV7_RNA_seq_statistics.csv |
| S10 | [RNA-seq data from a comparison of RMV7 epigenetic phase variants](https://figshare.com/articles/dataset/RNA-seq_data_from_a_comparison_of_RMV7_epigenetic_phase_variants/23589270) | RMV7_RNA_seq_JSDs.csv |
| S11 | [RNA-seq data from a comparison of RMV7 epigenetic phase variants](https://figshare.com/articles/dataset/RNA-seq_data_from_a_comparison_of_RMV7_epigenetic_phase_variants/23589270) | RMV7_RNA_seq_expression.csv |
| S12 | [RNA-seq data from a comparison of RMV7 epigenetic phase variants](https://figshare.com/articles/dataset/RNA-seq_data_from_a_comparison_of_RMV7_epigenetic_phase_variants/23589270) | RMV7_RNA_seq_expression.csv |
| S13 | [RNA-seq data from a comparison of RMV7 epigenetic phase variants](https://figshare.com/articles/dataset/RNA-seq_data_from_a_comparison_of_RMV7_epigenetic_phase_variants/23589270) | RMV7_RNA_seq_expression.csv |
| S14 | [Experimental data from the analysis of RMV7 phase variants](https://figshare.com/account/projects/171060/articles/23634165) | RMV7_qRTPCR.csv |
| S15 | [RNA-seq data from a comparison of RMV7 epigenetic phase variants](https://figshare.com/articles/dataset/RNA-seq_data_from_a_comparison_of_RMV7_epigenetic_phase_variants/23589270) | RMV7_RNA_seq_expression.csv |
| S16 | [Experimental data from the analysis of RMV7 phase variants](https://figshare.com/account/projects/171060/articles/23634165) | RMV7_motif_separation_expression_change_comparison.csv |
| S18 | [Experimental data from the analysis of RMV7 phase variants](https://figshare.com/account/projects/171060/articles/23634165) | RMV7_piuA_transformation_efficiency.csv |
| S19 | [Experimental data from the analysis of RMV7 phase variants](https://figshare.com/account/projects/171060/articles/23634165) | RMV7_motif_distribution.csv |
| S20 | [RNA-seq data from a comparison of RMV7 epigenetic phase variants](https://figshare.com/articles/dataset/RNA-seq_data_from_a_comparison_of_RMV7_epigenetic_phase_variants/23589270) | RMV7_RNA_seq_expression.csv |
| S21 | [RNA-seq data from a comparison of RMV7 epigenetic phase variants](https://figshare.com/articles/dataset/RNA-seq_data_from_a_comparison_of_RMV7_epigenetic_phase_variants/23589270) | RMV7_RNA_seq_expression.csv |
| S22 | [RNA-seq data from a comparison of RMV7 epigenetic phase variants](https://figshare.com/articles/dataset/RNA-seq_data_from_a_comparison_of_RMV7_epigenetic_phase_variants/23589270) | RMV7_RNA_seq_expression.csv |
| S23 | [RNA-seq data from a comparison of RMV7 epigenetic phase variants](https://figshare.com/articles/dataset/RNA-seq_data_from_a_comparison_of_RMV7_epigenetic_phase_variants/23589270) | RMV7_RNA_seq_expression.csv |
| S24 | [Experimental data from the analysis of RMV7 phase variants](https://figshare.com/account/projects/171060/articles/23634165) | RMV7_ciaRH_htrA_transformation_efficiency.csv |
| S25 | [Experimental data from the analysis of RMV7 phase variants](https://figshare.com/account/projects/171060/articles/23634165) | RMV7_rare_carbohydrates_transformation_efficiency.csv |
| S26 | [Experimental data from the analysis of RMV7 phase variants](https://figshare.com/account/projects/171060/articles/23634165) | RMV7_manLMN_growth_curves.csv |
| S27 | [Experimental data from the analysis of RMV7 phase variants](https://figshare.com/account/projects/171060/articles/23634165) | RMV7_manLMN_growth_curve_statistics.csv |
| S28 | [Experimental data from the analysis of RMV7 phase variants](https://figshare.com/account/projects/171060/articles/23634165) | RMV7_rare_manL_carbohydrate_transformation_efficiency.csv |
| S34 | [Experimental data from the analysis of RMV7 phase variants](https://figshare.com/account/projects/171060/articles/23634165) | RMV7_cAMP_concentrations.csv |
| S35 | [Experimental data from the analysis of RMV7 phase variants](https://figshare.com/account/projects/171060/articles/23634165) | RMV7_cAMP_transformation_efficiency.csv |
| S36 | [Experimental data from the analysis of RMV7 phase variants](https://figshare.com/account/projects/171060/articles/23634165) | RMV7_rare_nagA_tfoX_yjbK_growth_curves.csv |
| S37A | [Experimental data from the analysis of RMV7 phase variants](https://figshare.com/account/projects/171060/articles/23634165) | RMV7_rare_nagA_tfoX_yjbK_carbohydrate_growth_curves.csv |
| S37B | [Experimental data from the analysis of RMV7 phase variants](https://figshare.com/account/projects/171060/articles/23634165) | RMV7_nagA_transformation_efficiency.csv |
| S37C | [Experimental data from the analysis of RMV7 phase variants](https://figshare.com/account/projects/171060/articles/23634165) | RMV7_manL_nagA_expression_levels.csv |
| S38 | [Experimental data from the analysis of RMV7 phase variants](https://figshare.com/account/projects/171060/articles/23634165) | R6_manLMN_tfoX_yjbK_transformation_efficiency.csv |
| S39A | [Experimental data from the analysis of RMV7 phase variants](https://figshare.com/account/projects/171060/articles/23634165) | RMV7_pilus_growth_curves.csv |
| S39B | [Experimental data from the analysis of RMV7 phase variants](https://figshare.com/account/projects/171060/articles/23634165) | RMV7_pilus_biofilm_thicknesses.csv |
| S40 | [Experimental data from the analysis of RMV7 phase variants](https://figshare.com/account/projects/171060/articles/23634165) | RMV7_PRCI_growth_curves.csv |
| S41A | [Experimental data from the analysis of RMV7 phase variants](https://figshare.com/account/projects/171060/articles/23634165) | RMV7_clpPE_expression.csv |
| S41B | [Experimental data from the analysis of RMV7 phase variants](https://figshare.com/account/projects/171060/articles/23634165) | RMV7_rare_clpP_expression_with_CaCl2.csv |
| S42 | [Experimental data from the analysis of RMV7 phase variants](https://figshare.com/account/projects/171060/articles/23634165) | RMV7_heat_shock_growth_curves.csv |
| S43 | [Experimental data from the analysis of RMV7 phase variants](https://figshare.com/account/projects/171060/articles/23634165) | RMV7_chaperone_expression_with_CaCl2.csv |
| S44 | [Experimental data from the analysis of RMV7 phase variants](https://figshare.com/account/projects/171060/articles/23634165) | R6_CaCl2_titration.csv |
| S45 | [Experimental data from the analysis of RMV7 phase variants](https://figshare.com/account/projects/171060/articles/23634165) | RMV7_qRTPCR_CaCl2_expression_effect.csv |
| S46 | [Experimental data from the analysis of RMV7 phase variants](https://figshare.com/account/projects/171060/articles/23634165) | RMV7_heat_shock_hrcA_restoration.csv |
| S47 | [Experimental data from the analysis of RMV7 phase variants](https://figshare.com/account/projects/171060/articles/23634165) | RMV7_recombination_heterogeneity.csv |
| S48 | [Maximum likelihood phylogeny of TfoX N terminal domain](https://figshare.com/account/projects/171060/articles/23633988) | TfoX_N_terminal_domain.tre |
| S49 | [RNA-seq data from a comparison of RMV7 epigenetic phase variants](https://figshare.com/articles/dataset/RNA-seq_data_from_a_comparison_of_RMV7_epigenetic_phase_variants/23589270) | RMV7_RNA_seq_expression.csv |
